# Historical Sketch of the Progress of Medical Science, from January to July, Inclusive, 1819

**Published:** 1819-07

**Authors:** W. Hutchinson

**Affiliations:** London


THE LONDON

### Medical and Physical Journal.

1 OF VOL. XLII.] JULY, 1819. [NO. 245.

"For many fortunate discoveries in medicine, and for the detection of numerous "errors, the world is indebted to the rapid circulation of Monthly Journals; "and there never existed any work to which the Faculty in EUROPE and "AMERICA were under deeper obligations than to the Medical and Physical "Journal of London, now forming a long, but an invaluable, series." RUSH.

### *Historical Sketch of the Progress of Medical Science, from January to July, inclusive*, 1819.

"Δεῖν δέ μοι δοκέι καὶ ἶαυτ' ἐιδέναι, ὅσα τῳ ἀνθρώπῳ παθήματα ἀπὸ δυνάμιων ἔρχεται, καὶ ὅσα ἀπὸ σχημάτων.—Πολλὰ δὲ καὶ ἔσω καὶ ἔξω τõν σώματος ἐίδεα σχημάτων, ἃ μεγᾶλα ἀλληλων διαφέρει πρὸς τὰ πάθῆματα καὶ νοσέοντι καὶ ὑγιαίνοντι—ἃ δἒι πάντα ἐιδὲναι, ἧ διαφέρει ὅπως, τα ἀίτια ἐκάστων ἐιδὼς, ὀρθῶς τηροίης." HIPPOCRATES, περι αρχαιης ιητρικης.

SUCH were the sentiments of the great physician of nature, respecting the importance of anatomical acquirements to the perfection of physiology, as well as to a correct knowledge of the origin and nature of disease. When we duly reflect on this, and consider also the animation, or rather the exultation, with which he spoke of an opportunity he had of contemplating a human skeleton, we readily discern why those men who have most contributed to the improvement of medical science, have dedicated the early part of their lives to anatomical researches.

It was such sentiments, and the prospects they disclosed, that led JOHN HUNTER, BORDEU, SOEMMERING, VICQ D'AZYR, and BICHAT, to devote so much time to the tedious and health-destroying avocations of the dissecting-room, after it was supposed that WINSLOW and MONRO had carried descriptive anatomy to a state of almost absolute perfection. Their genius, to the character of which those labours on a slight view might appear so incongruous, enabled them to perceive that the most essential part even of this important knowledge, in regard to its application to physiology, was still to be acquired. Former travellers through this region, they saw, had furnished us with little more than such information as might be deduced from general and superficial observance, whilst that to be obtained from profound and accurate views still remained to be developed by the eye of the philosopher. The records of physiology will shew us the results.

Those who have a due sense of the importance of the truths above alluded to, will contemplate with much gratification the discoveries which have been made in that source during the late period; as, besides the interest they possess from their intrinsic value, they seem to shew, that there now exists an ardent spirit, the object of whose exertions is to render the present epoch that at which the history of the progress of descriptive anatomy may receive its final close.

In order to illustrate the latter observation most effectually, we should give an account of the discoveries which have been made respecting the brain and its appendages during the late period; but, as we shall in the ensuing Number of our Journal insert the first of a series of Memoirs on this subject, by Professor LAUTH, of Strasbourg, who has pourtrayed them in a very accurate and comprehensive manner, we shall pass over them on the present occasion with only a reference to the source whence a knowledge of them may be derived. (1)

§ 1. Commencing with the skeleton, our attention is first arrested by the researches of M. BECLARD respecting the *formation, increase, and senile atrophy, of the bones of the human species.* (2) But, before we proceed, it will be prudent in us to remark, that the view we give of anatomy will be but very partial and imperfect; as, in order to convey a clear and full idea of its subjects, it would be necessary to enter into minute and lengthened descriptions, that are incompatible with the plan of this essay. We cannot here by any effort seize at one grasp the principal ideas that form the bases of extensive arguments; and, by a few striking traits, give to a reflective mind the clue that might lead it through a whole region of knowledge. We shall, therefore, aim at another object; that of conveying sufficient information of the most interesting parts of the different subjects that come within our views; at the same time taking care to give to those who desire a more perfect degree of knowledge of them, a reference to the various sources whence our observations have been derived.

Recurring to the researches of M. Beclard, we find some observations respecting the periods at which ossification of the different bones usually commences, and the development of that process, which the consideration of their interesting nature, and their real practical importance on many occasions, lead us to select from those with which they are conjoined in the original memoir. Ossification commences in the human fœtus about thirty days after conception, and in the following order: In the clavicles, inferior and superior maxillary bones, the humerus,

(1) *Memoire sur la Structure du Cerveau et de ses Annexes.* Jour. comp. du Dict. des Sciences Med. t. iii.

(2) *Memoire sur l'Ostéose, ou sur la Formation, l'Accroissement, et l'Atrophie Sénile des Os dans l'Espèce Humaine.* Nouveau Journal de Medicine, t. iv.

femur, radius, cubitus, and the tibia. The points of ossification at the age of five weeks, were about a line and a half in length in the clavicle.

The spine, which in the adult is about two-fifths of the whole length of the body, bears a different proportion to it at different ages. After the lapse of three weeks of intra-uterine life, when the embryo is about four lines in length and its limbs pointing like buds, the spine is in the proportion of three to four of the whole body. At the age of thirty-five days, the length of the fœtus is from 12 to 18 lines, and the proportion of the spine is as 3 to 5; at from forty to forty-five days, length of the fœtus from 24 to 30 lines, the proportion of the spine as 1 to 2. At two months, the whole length is about 4 inches and 3 lines, that of the spine 2 inches. At three months, the former is 6 inches, and the proportion of the spine as $2\frac{2}{3}$ to 6. At four months and a half, it is 9 inches, and the spine 4; at six months, 12 inches, the spine 5; at $7\frac{1}{2}$ months, 15 inches, the spine $6\frac{1}{3}$; at nine months, or the period of birth, the fœtus is ordinarily from 16 to 20 inches in length, or at a medium, 18; and the spine is in the proportion of $7\frac{3}{4}$ to 18 to the whole length of the body. These calculations were made from observations on about fifty fœtuses, at each of the above-indicated periods.

Ossification commences in the bodies of the vertebræ between the fortieth and forty-fifth days; it had begun in their apophyses a few days previously.

Each vertebra, consisting originally of a section of a solid cylinder, and a ring furnished with several apophyses, is in general formed by three primitive points of ossification: the one anterior, which, by its development, forms the body or solid part of the bone; and two lateral ones, which constitute the apophysarial masses, and which uniting together and with the former constitute the annular structure. Besides these, each vertebra is completed by several secondary points of osseous development.

At about the sixth month of intra-uterine life, two points of ossification are found in the second cervical vertebra, one situated above the other. Towards the seventh month, the superior point, which answers to the odontoid process, is larger than the inferior, which relates to the body of the bone. At about the eighth month, the transverse processes have begun to ossify in the first of the lumbar vertebræ. At the time of birth, ossification has commenced in the body of the first cervical vertebra, and also in the first bone of the coccyx. At this age, the body of the fourth lumbar vertebra, which is the most voluminous, is three lines in depth and six lines in breadth. At the same period, the lateral portions of the six superior dorsal vertebræ begin to unite together, so as to form a ring posteriorly to the bodies of those bones. The lateral arch of the second, which is the largest, forms a chord of seven or eight lines. At the age of one year, those processes are united, so as to form a ring in all except in the first two cervical, and in the lumbar, vertebræ; and in the bones of the sacrum. At two years and a half, the union is completed in the transverse processes of the first and second cervical, in the last of the lumbar vertebræ, and in the inferior bones of the sacrum. At five or six years of age, the whole have perfectly united.

Merely pointing out one instance of the utility of their application, we may remark, that the facts last mentioned (many of which, however, were previously known,) will explain the peculiar appearance of *spina-bifida*, and the reason why it usually occurs in the situation of the lumbar vertebræ. They will shew why the tumor containing a fluid should appear in this part, when the disease on which it depends is often seated in the brain, or throughout the whole extent of the spinal marrow.

The observations of M. Beclard on the formation and developement of the cranium, possess a great degree of individual interest; but we pass them over in consequence of this part of the subject having been pursued to a greater extent by Hr. J. F. Meckel, of Halle, whose Memoir (3) on it is now to become the object of our attention.

These researches were, apparently, instituted in consequence of some curious ideas deduced from observations made in comparative anatomy; and they seem to have developed a physiological principle that may be applied with considerable benefit in the practice of medicine. It is this: that the vertebral column and the cranium perfectly correspond with the organs they enclose, not only with regard to form, but also in the manner in which they are developed. As the vertebral column is formed of annular or discoid bones piled on each other, so we may represent to our mind the spinal marrow constituted of spherules of nervous matter, from each of which is derived a pair of nerves, and each of which corresponds with an individual vertebra: the number of the pairs of nerves also equals that of the vertebræ, and each pair escapes from the vertebral canal between two of them. Each segment, as well of the bony column as of the nervous mass, possesses its proper vascular system; and as the vertebræ are chiefly formed of two lateral portions, so is the spinal marrow composed of two longitudinal cords applied laterally to each other.

There exists between the cranium and the encephalon, the same relation that we have noticed as existing between the vertebral column and the spinal marrow. The bones which

(3) *Journ. comp. du Dict. des Sciences Med.* tome ii.

compose this extensive vase correspond in their position, their division, and the mode of their development, to distinct portions of the organ which they enclose. We cannot enter into a particular account of the observations of Hr. Meckel in support of this statement, but we must remark, that he appears to have satisfactorily demonstrated its truth. This distinguished anatomist supports a curious proposition, which OKEN advanced above ten years since, and which M. Beclard has also illustrated, that there is a similarity or relation between the different bones of the cranium and the vertebræ: a certain division of each corresponding with a distinct portion of the other, both in the form and the mode in which they are developed.

This notion appears to be a further prosecution of some curious ideas lately advanced by several eminent anatomists respecting the origin of the brain, which are now generally admitted in Germany,—that it is a production of the spinal marrow; (4) and the reflections to which this opinion gives rise have probably contributed much to raise up the doctrines respecting the cerebral functions now prevalent in that nation. We shall not expressly point them out, for reasons which those who perceive what we allude to will easily recognize; but our duty as an historian obliged us to relate the observations which led to this remark.

Hr. Meckel in the same memoir enters into a particular consideration of the mode in which the whole of the osseous structure surrounding the three centres of the nervous systems is developed; but we cannot adduce any further observations which, in an isolated point of view, would be interesting to the reader.

We can only speak in general terms of a Treatise by M. GEOFFROY SAINT-HILAIRE, on the *anatomy of the osseous structure of the respiratory organs.* (5) This work will be perused with extreme pleasure by naturalists as well as by physiologists. The author has elucidated, in a very clear and ingenious manner, a law in the economy of nature which is again becoming generally admitted by philosophers: that the animal powers and functions are developed in proportion to the wants of the individual; or in the words of an Epicurean,

> Nihil ideo quoniam natum est in corpore, ut uti
> Possemus; sed quod natum est, id procreat usum.

M. St.-Hilaire also proves, with regard to structure, that in

---

(4) For a general view of this subject, see C. G. CARUS; *Versuch einer darstellung des Nervensystems, und insbesondere des Gehirns, nach ihrer bedeutung entwickelung und Vollendung im thierischen organismus.* Leipzick, 1814.

(5) *Philosophie Anatomique des Organes Respiratoires, sous le rapport de la determination et de l'identité de leurs pièces Osseuse;* par GEOFFROY SAINT-HILAIRE. 8vo. pp. 560; avec un atlas in quarto de 10 planches. Paris.

all vertebrated animals nature has followed a general plan, some points of which have only been varied in the different species; and that the passing from one form to another, in similar organs, is effected only by almost insensible gradations.

This proposition appears to have been first developed and illustrated by Spix; (6) although it was probably founded on the views which Oken (7) had taken of the analogy existing between the spine and cranium.

Those observations, with several other original and ingenious propositions, are elucidated in a very forcible manner by M. St.-Hilaire, whose work, though confined to only a few objects, constitutes an highly valuable addition to modern literature on natural history.

M. Serres has been also engaged in researches on this part of descriptive anatomy. A Memoir which he lately presented to the Academy of Sciences of Paris, on *the laws of osteogeny*, treating especially on the formation of the articular cavities, evinces the same accurate and ingenious views in this enquiry that gave so interesting a character to his former productions. But we may avoid the necessity, and indeed the utility, of giving a particular account of his observations, by referring our readers to the papers of Mr. Howship on the same subject, in the eighth and ninth volumes of the Medico-Chirurgical Transactions, who has prosecuted it in a more particular and extensive manner, and on similar views with those which guided M. Serres.

We never contemplate the productions of the learned and ingenious Meckel, without regretting that he was not a compatriot of, and contemporary with, our Hunter: the latter would not then have been doomed to pursue his researches silent and alone, unknown to the world except by their results, and those for a long time undervalued, because their merit was not discerned: he would have cherished with delight the genius of the German philosopher, whose talents and society would have assisted and cheered his solitary labours.

We shall adduce only a few from amongst a multitude of analogous observations, contained in a Memoir by Professor Meckel, (8) on *the formation of the intestinal canal in the mammiferi, and particularly in man*, which will show those who imagine minute anatomical research to be dull and useless, how delightful it becomes when directed by physiology, and how many facts may even now be discovered which will illustrate pathology, and consequently contribute to the improvement of the medical art. The first series of extracts will elucidate the cause

---

(6) *Cephalogenesis*, &c. Munich, 1815. fol.

(7) *Uber die Bedeutung der Schaedelknochen.* Jena, 1807. 4to.

(8) *Journal comp. du Dict. des Sciences Med.* tome ii.

of the frequent appearance of umbilical hernia in the fœtus, and in the infant at the period of birth.

"The intestinal canal," says Hr. Meckel, "presents many changes in its situation at different epochs in the life of the fœtus. Although in its origin it is attached to the vertebral column, and is at first entirely straight, it is certain that, at a more advanced period, (which is, however, still very near the instant of its origin when compared with the whole life of the fœtus,) it is more separated from it, relatively speaking, than it is at any subsequent period during the whole course of its existence; that is to say, it passes out of the abdominal cavity in the place which is afterwards to become the navel, and, passing through the opening there observed, it is engaged in the umbilical cord." "I have always found a portion of the intestinal canal engaged in the funis umbilicalis, until the commencement of the third month, (meaning, after it has separated from the spine,) although the embryoes that evinced this appearance did not shew the smallest trace of irregular conformation in any other point of their organization." "In a great number of the mammiferi, especially in the goat, the sheep, the cow, the pig, and the rabbit, I have always found, at a certain period, the greatest part of the intestinal canal in the umbilical cord. The same fact has been observed by Emmert, Hoechstetter, and Paletta."

The correctness of these observations is, however, disputed by Osiander: (9) he says, that in all cases where this projection of the intestinal canal from the cavity of the abdomen is witnessed, and at whatever period it may occur, it is to be considered as a state of disease. But we rely on the extent and accuracy of the observations of Hr. Meckel, and are disposed to admit the occurrence of the fact in the manner in which he has stated it.

"At first," says this anatomist, "we only see springing out a small portion, forming an acute angle; but this by degrees increases in bulk, and forms several convolutions. At the epoch when these convolutions manifest themselves, we see the coecum appear in the form of a little tubercle, with its extremity pointing anteriorly: it is situated on the inferior side of the angle, and never on the angle itself." "By degrees the circumvolutions approach each other, the hernial portion is converted into a more rounded projection, situated immediately within the umbilical opening: this gradually contracts, and the cavity of the cord which contains the hernial portion of the intestine becomes separated from the abdominal cavity by a more pronounced contraction. The intestine insensibly re-enters into

(9) Salzburger *Medicinisch-Chirurgische Zeitung.* No. 89. 1814.

the belly, the colon passing first, and the small intestine afterwards." The meso-colons after this gradually contract, until they assume nearly the proportion they afterwards bear to the dimensions of the relative organs.

It is evident that the accidental want, or later occurrence, of those various changes in the structure of the parts above described, will cause the appearance of umbilical hernia at the time of birth; and we cannot be surprised at its frequency, when we contemplate the nature of the process by which it is ordinarily prevented. The same observations, especially the gradual contraction of the meso-colons, will explain why, with a few exceptions, those hernia at length spontaneously disappear.

We shall now adduce some extracts respecting the development of the structure of the alimentary canal appertaining to the exercise of its proper functions.

"The villosities of the stomach appear at a very early period," observes Hr. Meckel, "but they pass through several degrees of evolution which merit being remarked. It is at the commencement of the third month that I have begun to perceive them distinctly. The internal surface of it is then rendered unequal by several longitudinal folds, strongly marked, pressed against each other; but of which, however, the free part is hardly rugous. By degrees, the number of these folds, their depth, and the quantity of rugæ, increase, so that at the end of the fourth month, sometimes earlier, we find, instead of simple longitudinal folds, a multitude of little elevations disposed without apparent regularity. The intestinal villosities, then, derive their origin from the rugæ, which manifest themselves gradually and in progression in the simple longitudinal folds. Another circumstance worthy of remark, is, that we find them at first much more diffused than they are at a subsequent period. Indeed, they are met with throughout the whole intestinal canal until the seventh month. The valvulæ conniventes appear much later than the villosities. Not the least trace of them is found until towards the seventh month. They then appear under the form of slight elevations, which disappear with great facility when the canal is a little stretched. The canales choledochus and pancreaticus, are always separate in the first instance. The pancreatic canal opens by a round tubercle into the left side and upper part of the descending portion of the duodenum. The orifice of the canalis choledochus is situate much lower, and on the right side of the intestine. The two orifices are much larger than they are at a subsequent period; and they gradually become united: this occurs soon after the middle of the third month. I have never seen the smallest trace of the pylorus before the expiration of the fourth month."

"The projection of a valvular form, which the pylorus produces in the duodenum, is very slowly developed: it is hardly perceivable at the age of six months, and it is still, speaking in a relative manner, very slightly evident in the fœtus at birth." "I have been struck with the state in which I have found the internal membrane of the stomach until the commencement of the fourth month. Before this period has elapsed, it has appeared not only thicker than in the adult with respect to the others, but also entirely separated from these, so that it formed a complete and perfectly free sac. It cannot be doubted but that this separation was the consequence of death; but it shews that the membranes are very feebly united during life."

The above are only some few, and dispersed, observations, amongst the very interesting series that are related in this memoir; but the whole occupy nearly fifty pages, and it is impossible to give a connected view of them in an abstract. They also lose much of their importance on being detached from the illustrations from comparative anatomy with which they are accompanied in the original.

The extent to which we have indulged our inclinations in the adducing of the foregoing extracts, prevents our doing any thing more than give a reference to some *observations on the different varieties noticed in the distribution of the brachial artery,* (10) by the same anatomist. The value of these is less determinate, since the circumstances to which they relate are too vague to admit of descriptions applicable in a general manner.

*A memoir on the formation and connexions of the crural arch, and other parts concerned in inguinal and femoral hernia,* (11) by Mr. Liston, of Edinburgh, is now to engage our attention. The great variety, the author observes, which exists in the descriptions of these parts given by different authors; the difficulties which students find in comprehending the subject; and the facility with which his own pupils were made to understand it by his description, were amongst the motives that induced him to publish these observations. With respect to the mode in which this is accomplished, none but a student can form a correct judgment; since what may appear lucid to one already acquainted with the subject, may be very obscure to those who have yet to attain this knowledge. But, as we do not think that indolence in the student, and an aversion to the use of the scalpel, should be encouraged, we shall proceed to adduce the other reasons which inclined Mr. Liston to publish this memoir. He says,

(10) *Loco citato,* tome iii.
(11) 4to. pp. 22, with 3 plates. Hill and Co. Edinburgh; and Baldwin and Co. London.

"It is one of my objects to shew, that the ligament of Gimbernat is totally unconnected with that of Poupart; that this part, or, what is the same thing, the inferior pillar of the external ring, instead of being turned down from its attachment to the tuberosity of the pubis, to be fixed to its crest, frequently sends fibres, in a curved direction upwards, to strengthen the conjoined tendon of the internal oblique and transversalis. The ligament of Poupart has also been named the crural arch; and the different fasciæ, in this situation, have been described by some as attached to its upper or lower edge; by others, as arising from it. It will however appear, that the crural arch is formed by a union of the fasciæ, under the ligament of Poupart: and this we shall proceed to shew."

The account of Mr. Liston has not convinced us of the erroneousness of the notion he opposes; but it has induced us to think that the cautions given by the most eminent and experienced anatomists, not to consider every portion of condensed cellular membrane as a distinct fascia, has not been sufficiently attended to. The interesting nature of the subject should, nevertheless, induce the practical surgeon to devote an hour to the perusal of the work.

§ II. Pursuing the progressive path of zoology, we now arrive at the station from which we are to take a view of some of the phenomena of the vital functions of man. On contemplating the beauty and order of the scene before us, and considering that its present appearance is chiefly the result of the labours of less than a century, it is not without sentiments almost of admiration that we regard the powers which have effected so interesting a change; and, looking round for the discovery of them, it is evident, that, although the full importance of the mode of philosophising instituted by Bacon and Locke, and perfected by Condillac, be admitted, especially that of the axiom of the former, reprehending "the easy passing over of the cause of things, by attributing them to secret and hidden virtues and properties, which had arrested and laid asleep all true enquiry and induction;" still it is evident that but little of what is now witnessed could have been effected by any other exertions than those directed by the hand of anatomy.

It was not until Willis, Malpighi, Whytt, Haller, Glysson, and Bordeu, had investigated the intimate structure of the different organs, especially that of the elementary tissues, that much precise knowledge of the laws of physiology had been acquired. But, notwithstanding their labours, and even the researches of John Hunter, although the functions of several organs, individually considered, were somewhat accurately understood, the laws which regulate the harmony of the whole were yet to be developed. There, we should probably have long remained, but for another train of enquiry, which appears to have been commenced as early as the latter part of the seventeenth century by CHIRAC, (12) and of which the importance was first clearly shewn in England, by JAMES JOHNSTONE: (13) we mean experiments on living animals. It is to these that we must in a great degree attribute the late progress of physiology; which has been such as will fully vindicate the conduct of the philosophers who instituted them, against the declamatory objections of those who, unacquainted with the recent history and present state of this branch of science, affect to contemn the knowledge which has thence been acquired.

We have been led to make the latter remarks, in consequence of those objections having been lately renewed by a person whose character and authority might have such an influence on the minds of some of the younger students in medicine, as would lead them to neglect the acquisition of what they will, on due application and study, find to be of the highest importance in the practice of the healing art. We shall proceed to the express object of this history.

In the arrangement of our observations on such works on physiology as will engage our attention, it is proper that those which relate to general treatises should be first adduced; and then such as appertain to more partial dissertations or individual remarks. We commence with the work of Sir THOMAS C. MORGAN. (14)

Some circumstances of an extraordinary nature which have recently occurred, render it prudent in us to adduce some adventitious remarks, before we proceed to the especial consideration of the work now before us. We allude to the public clamour that has lately been raised (chiefly, we should observe, by persons who have shewn themselves not qualified to pass a judgment on the subject; which is, in reality, totally without their province,) against some of our physiologists, who have published doctrines founded on materialism respecting the nature of the vital functions and intellectual faculties of man. This clamour is extraordinary, because the work of HELVETIUS *de l'Esprit* is to be found in every library translated into our language, where it has peaceably remained for half a century. Now, it is hardly possible for the doctrines of materialism to be more openly declared than they are in this work, and as little possible for them to be displayed in a more captivating manner. Why, then, the publishing of the same doctrines by some of our

(12) See *Journal des Sçavans*, année 1688. p. 36.
(13) See *Philosophical Transactions*, vol. 54, anno 1754, and vol. 57.
(14) *Sketches of the Philosophy of Life*; by Sir T. C. Morgan, M.D. Fellow of the Royal College of Physicians, of London. 8vo. pp. 466. Colburn, London.

own writers should be considered so outrageous to propriety, we are utterly at a loss to comprehend. If the original advancement of them be criminal, surely the study of them should not, as is the case, be publicly permitted, and even encouraged, in the chief of our public schools. However, those circumstances oblige us to point out in an express manner, the view in which we consider the work of Sir Thomas Morgan, when we speak of it as possessed of a high degree of merit as a treatise on physiology.

We in the first place observe, although it may be considered superfluous, that researches after the proximate causes of natural phenomena, are now generally acknowledged to be vain and futile efforts of the intellectual faculties of man: this is a truth that has sooner or later been discerned by every nation where the understanding has been well cultivated, and the powers of the senses correctly appreciated. It is this which is shown by the Egyptian symbol of the veiled Isis, pointing to the expressive sentence—

Τον εμον πεπλον ουδεις πω θνητος απεκαλυψεν; it was discerned by the most wise and virtuous sect of the Grecian philosophers; and has constituted the first axiom of those who have most contributed to effect the late progress of physical science. Attempts made to explain what may be obscure in natural phenomena by springing from the route of precise observation, will more probably lead us into some of the numerous paths of error, than into the single one of truth. This, we repeat, has for several years been a ruling axiom in the principles for the conducting of physical researches; several eminent philosophers have equally applied it to those relative to the physiology of man; and it has been assumed by Sir Thomas Morgan in the treatise now before us. The author views in the phenomena of mind, as well as in those ordinarily termed, expressly, the animal functions, the results of a certain mode of organization, composed of matter, and regulated by laws, respectively identical with, and analogous to, those which produce the general phenomena of nature; excluding from his arguments the consideration of all agents which are not visible to the senses.

"There may indeed," he says, "exist in *rerum natura* forms of matter, whose properties stand not in relation to our senses, and whose affections and modes of action we cannot conceive; but, for every purpose of reason, such substances are to man as if they had no existence. (15) They can form no part of his

(15) "Il n'existe pour nous de causes exterieurs que celles qui peuvent agir sur nos seus, et tout objet, auquel nous ne saurions appliquer nos faculties de sentir, doit être exclu de ceux de nos recherches." CABANIS. See also LOCKE on the Extent of Human Knowledge, § 6.

system of knowledge, nor can they be made to harmonize with material agency. As far, therefore, at least, as ideas are the subjects of human contemplation, they must be regarded as changes impressed upon the substance of the brain, by the impact of bodies that are external to its tissue. To this point alone can investigation be satisfactorily conducted. Enquiries pursued beyond organization terminate only in the wildest conjectures, and the most contradictory propositions; whilst the reduction of intellectual action to the same laws as those which govern other organic phenomena, affords a positive and satisfactory base for moral and metaphysical investigation. (16)

"The admission of this fact has no relation whatever with the doctrine of an immortal soul, nor with any dogma, founded on faith, and independent of reason. (17) The theological soul must be clearly distinguished from the *primum mobile* of relative sensibility; in order to avoid the obvious dilemma of conferring immortality, not only on animals but on vegetables, or, on the other hand, of denying it to man." (18)

Considering this work and its objects in the way in which the author here states that it should be regarded: that is, merely as a view of the phenomena of life, without insisting on what may be its proximate cause; and neglecting, therefore, the question whether or not there be added to material organization, the νȣς and ψυχη, the animus and anima, or the vital principle and soul: in other words, viewing it as a Treatise on Physiology, in the ordinary sense of that term, we must confer on it our warmest approbation, and place it amongst the most valuable literary productions of the present period. We ought, also, to recommend it to the attention of medical physiologists; since, apparently from the epigraph with which it commences, (19) an idea is commonly entertained that it is solely, or principally, calculated for the use of the student of general philosophy; wanting that depth and precision of information that are required by the physician. This idea is erroneous. These sketches are deduced from very extensive and accurate observ-

---

(16) That thought consists in movements, and is consequently obedient to the general laws of motion, is evident from the single fact, that time is necessary for its accomplishment.

(17) "These dogmas rest entirely upon scripture authority; and the soundest divines have refrained from mingling philosophical investigation with religious faith." See *Memoirs of Bishop Watson*, on the *Locality of the Soul*, vol. i.

(18) "But here I take the liberty to observe, that, if your lordship allows brutes to have sensation, it will follow either that God can and doth give to some parcels of matter a power of perception and thinking, or that all animals have immaterial, and consequently, according to your lordship, immortal souls, as well as men; and to say that fleas and mites have immortal souls, as well as men, will possibly be looked upon as going a great way to serve an hypothesis." LOCKE's *Letters to the Bishop of Worcester*.

(19) Χρὴ πάντας ἀνθρώπους ἰητρικὴν τέχνην ἐπίσlασθαι, ὦ Ἱππόκρατες, καλὸν γὰρ ἅμα καὶ ξυμφέρον ἐς τὸν βίον.

ance, and the most profound scientific as well as literary erudition; and, however much we may be pleased with the elegance and grace of the style and manner in which they are written, we most approve the severe logical accuracy with which the arguments are developed. With respect to its medical doctrines, distinctly considered, we should adduce but few objections, and those not on points of much importance, were we to pass over it a particular critical review. The parts of it with which we have been most pleased, are those on the elementary tissues; the remarks on the theories of the proximate principles of the fluids, &c.; assimilation and secretion in general; the remarks on the theories of respiration, the particular secretions, and the general and particular laws of vital action. The observations on disease in general, though concise, disclose a lucid view of the modern and approved doctrines of pathology. There are but few persons who may not receive useful information from this work, and none, we hope, who will not derive much gratification from the perusal of it; and to those, especially, who have not kept pace with the recent progress of medical science, it will prove a highly valuable acquisition.

As the sketches of Sir Thomas Morgan are calculated to lead the reader to metaphysical speculations, especially as connected with the physiology of man, we earnestly recommend to the student who may be disposed to pursue them, a recent work by an excellent metaphysician and moralist, (20) in which the subject is treated, in this point of view, in a very lucid and comprehensive manner, and with an object which will be best shewn by the author himself, in the following paragraphs:

"Dans le premier livre nous parlerons de l'Etre proprement dit; le suivant traitera du néant qui sera à sa place, puisque la création en est sortie; nous entretiendrons ensuite le lecteur a l'être matériel; de l'être vivant, sensible, intelligent, et moral; de leur union, de leur separation, et du rétablissement nécessaire de tous les deux dans l'espèce perfectionnée.

"Nous osons nous promettre que nul ne sortira de la lecture de notre livre avec un sentiment moins profond des perfections divines, qui celui avec lequel il l'aura commencée. Nous ne craignons pas d'affirmer que nourrissant des idées plus nobles de sa propre nature, il sera porté à respecter davantage en lui-même l'être réservé aux grands desseins que nous lui aurons fait entrevoir."

Pursuing those benevolent and judicious intentions, M. Keratry appears to have endeavoured to conciliate the ideas of Plato respecting the Deity and the soul of man; those of

(20) *Inductions Morales et Physiologiques*, par M. Keratry, 8vo. pp. 451, Paris, 1818.

Locke and Condillac on the human understanding; those of Pascal, La Rouchefoucault, and Helvetius, on the determining principles of virtuous and vicious actions; and, lastly, our knowledge of zoology and physiology, with the fundamental principles of the established religion. This work is the result of much original observation, very extensive researches, and long and judicious reflection; and, combining its scientific value with the moral doctrines it inculcates, it must be considered as a very interesting and important addition to modern literature.

We pass over in silence one work in our language, in which these elementary principles of physiology are also discussed, from reasons which we cannot reflect on without sensations of deep regret. We need not expressly designate it, for it will be recognized by our readers; yet, we could not avoid alluding to it in an historical record of works conducive to the improvement of medical science.

The next in order, with respect to the general extent of the views it discloses of physiology, is a treatise calculated to excite much interest amongst the more reflective part of medical enquirers; as being that of a physician of the first eminence with regard to professional character, and well known to be possessed of a philosophic disposition of mind: this is the *Elements of Medical Logic*, (21) by Sir GILBERT BLANE. We speak of it in this place, since physiological illustrations of those Elements constitute the principal part of the work. These are views of the author, "quæ retenta animo, remissa temporibus, longo intervallo intermissa revocavit;" and, contemplating them thus, they are too important to be treated in a general manner: we therefore defer the consideration of them to another occasion, when, after having passed in review several important works more immediately of practical importance, we can with more propriety indulge in the extensive exposition of them which we are disposed to adduce.

We now take up a work (22) by Dr. CHARLES HENRY PARRY, the chief object of which is to support a point of doctrine of the distinguished author of the "Elements of Pathology;" that is, the passiveness of the arterial system in the circulation of the blood. We should observe, previously to the consideration of this subject, that the occurrence of union between the extremities of a large artery divided by a ligature, by means of small arteries passing from one to the other, (a fact first shewn to

(21) *Elements of Medical Logick, illustrated by practical Proofs and Examples; including a Statement of the Evidence respecting the contagious Nature of Yellow-Fever.* 8vo. pp. 219. London, 1819. T. and G. Underwood.

(22) *Additional Experiments on the Arteries of Warm-blooded Animals; together with a brief Examination of certain Arguments which have been advanced against the Doctrines maintained by the Author of "An Experimental Enquiry, &c."* By Charles Henry Parry, M.D. F.R.S. &c. 8vo. pp. 257. 1819. Longman and Co.

take place by the author of the "Inquiry," &c.) is proved by repeated experiments instituted by Dr. Charles Parry. The same appearance was also observed in the animals which were stated to be under the process of the experiment at the time of the publication of the "Inquiry."

The doctrine of that part of the present work involving the question, whether or not the arteries contract and dilate under ordinary circumstances consonantly with the pulse, should now be examined. We commence with an extract.

"It has been imagined, that if any power, whether of contraction or dilatation, or of both, can be established as existing in these vessels, especially in cases where the heart can be supposed to have little or no influence, the conclusion is logically accurate, that, in ordinary states of the circulation, the arteries assist the heart, and promote the course of the blood.

"It is singular that so much unnecessary pains should have been taken by men of talent, to prove what has never been denied; nay, to prove positions which the author of the 'Inquiry' has himself attempted to establish as the basis of his own pathological doctrines. We shall discover in all his writings ample evidence of his conviction, that arteries have very considerable powers in themselves; that they are capable of dilatation and contraction; that they may experience either or both varieties, by the influence, or independently, of the heart's action; that there are various independent causes of motion, distinct from those which belong to this organ, and existing even when its influence is wholly destroyed. These positions were, indeed, at once admitted and illustrated by him; but the sweeping inference must still be rejected, that such an admission can, by any construction, prove the efficacy of these causes in producing the state of undisturbed circulation under the ordinary circumstances of life."

Passing over the change, or at least extension, of the face of the question given in the last paragraph,—for the admissions there stated were not made in the "Inquiry," (23)—we observe, that if the facts implied in those admissions do not *prove* that the arteries, by contraction and dilatation, aid the heart in the circulation of the blood, under ordinary circumstances, they at least render this highly probable by the most severe analogical reasoning: and, if this sort of testimony be not admitted, the present system of physiology must be considered a baseless fabric; for the greater part of it rests on no firmer foundation. The facts principally alluded to in the above paragraphs, are cases analogous to those of which we shall now refer to instances: and as the most forcible, those where the left ventricle of the heart

(23) This is the interpretation of all those who engaged in the dispute.

has been changed into a firm osseous, or petrous, matter, (24) "en une véritable pétrifaction;" and another where the left ventricle was converted into fat. (25) In these cases, the circulation was vigorously carried on. In the former, the pulse at the wrist "avait de l'élévation," and "le gauche ne différait nullement du droit." The patient of the latter died in a fit of apoplexy, from serous effusion. In this case, the pulse was irregular for some time before death. Now, it is not rational to suppose that the arteries could carry on the circulation in the manner in which it was done in these cases, and yet not contribute to effect it under ordinary circumstances. There is no decided instance of a part of the animal economy performing, in so perfect a manner, a function which it does contribute to effect under ordinary circumstances. It is an established axiom, amongst the best naturalists, that functions are the consequences of wants: how a function could be so perfectly fulfilled, and the want not essentially exist at the time of the formation of the body, is utterly inexplicable. But Dr. Charles Parry, when speaking of somewhat analogous occurrences, says,

"It might seem unnecessary to repeat, that the argument drawn from akerious fœtuses, akerious animals, and from the circulation in the egg before the heart is perfected, is adduced *ad ignorantiam;* we may however add, that these and all similar objections, if any thing, are more than is wished; as the most regular inference from such facts is, not that the heart and arteries have conjoint powers, but that the heart is not necessary for the carrying on of the circulation of the blood."

And in another part of his work, he observes,

"Other arguments in favour of the arterial power, as aiding the circulation, are taken from their supposed dilatation and contraction; from their muscular fibres; from the effects of diseased vessels; from the violent hæmorrhages occurring from inflamed arteries; from the violent effects of a vessel ruptured in the head; from the throbbing of all the large arteries of the arm, as far as the axilla, from abscess at the end of the finger; from the pulsation of the carotids and the abdominal aorta; from the phenomena of blushing; from the retrograde and oscillatory motion of the blood; from the powers of the vena portæ

---

(24) See *Journal de Medecine*, Jan. 1806; or *Essai sur les Maladies du Cœur*, par M. Corvisart. It may be proper to remark, that this case is related by M. Renauldin. Mr. Allan Burns mentions a case where "the whole extent of the pericardium covering the ventricles, and the ventricles themselves, except about a cubic inch at the apex of the heart, were ossified and firm as the skull." Mr. Charles Bell has in his collection, specimens of a similar change of structure, though not to so great an extent.

(25) *Dublin Hospital Reports*, vol. ii. See, in the present "Sketch," the section on *Pathological Anatomy*. This case is related by Dr. Cheyne.

as a secreting vessel; from the state of the vessels during the growth of parts, &c. &c. From the admission of these and many other similar examples of an action apparently unconnected with the heart's impulse, it is supposed, that in ordinary states the arteries must also be admitted to assist the heart. This principle, however, cannot be conceded. We may, perhaps, still further deny that all these instances are proofs of a peculiar independent power, that they all have existence, or that they must inevitably produce the effects which are attributed to them."

Really, this appears to be similar to the conduct of Democritus respecting the sweet cucumber: and what is there to oppose to the notions which the above, and many other apparent facts equally forcible, decidedly favour?—Merely the want of evidence to the senses, of such an occurrence in experiments made on brute animals.

We cannot of course in this sketch enter either into a regular disputation on this question, or a critical examination of the work before us; but it is a part of our duty as an historian to state the opinions we hold respecting its doctrines: we therefore say, that, although dilatation and contraction of the arteries concurring with the heart to effect the circulation of the blood, does not appear to be *proved*, it is forcibly inculcated by what must be considered an exact mode of reasoning. We observe that the tone of the statements on the opposite side of the question is somewhat different in the work of Dr. Charles Parry, from what it was in that of the author of the "Elements of Pathology," (we like thus to designate him.) It is particularly evident in the latter part of the work, and the author concludes in the following manner:

"The observation of SCARPA still continues applicable to the present state of facts, and with it I shall conclude this imperfect essay. 'L'observation et l'expérience ne vont pas encore jusqu'a prouver pour les esprits exactes, que les grosses artères, et même celles du second et du troisième ordre aient des fonctions actives à remplir dans l'acte de la circulation. L'induction n'a prouvé le contraire que pour le systême capillaire.'"

Mr. CHARLES BELL, in an essay (26) on this subject, advances, with much confidence, opinions on the opposite side of the question to that supported by the authors of the "Inquiry" and the "Additional Experiments." He says,

"When we reflect that the blood of some creatures circulates

(26) *An Essay on the Forces which circulate the Blood; being an Examination of the Difference of the Motions of Fluids in living and dead Vessels.* By CHARLES BELL, F.R.S. E., Surgeon to the Middlesex Hospital, and Lecturer on Anatomy and Surgery in Great Windmill-street. 12mo. pp. 83. 1819. Longman and Co.; and Burgess and Hill.

without a heart, and see acephali born without a heart, yet fully nourished; and when we see the aortic system of fishes removed almost out of the influence of the heart; and when we see that the heart of all animals is placed in juxtaposition, and in accurate sympathy, with the lungs; it is impossible to refuse assent to the proposition, that the arteries possess the chief power in circulating the blood through the corporeal system; and that the heart is rather the regulator than the prime and efficient cause of the circulation. And by this it is not only meant that its state of excitement and activity commands and draws after it the motion of the blood generally, but that it regulates the actions of the lungs, in exactly according with the state of the blood and the necessities of the system." (27)

Mr. Bell reasons in favour of this opinion from the structure of the arteries, from various mechanical experiments, and from physiological and pathological phenomena of almost constant occurrence. But we shall not adduce his arguments, since similar, or nearly analogous, to the chief part of them, had already been advanced by GALEN, WHYTT, SENAC, DUMAS, BOERHAAVE, STAHL, SOEMMERING, WILSON PHILIP, and others. Those which relate to physiology and pathology especially, were fully developed by BORDEU: (28) indeed, no other author has more clearly and beautifully illustrated the subject in this point of view; and it is very extraordinary that his arguments have not been noticed in the discussion. But his works show the truth of the remark of BACON,—that the course of science is like a rapid stream, which brings down to us such light things as float on its surface, whilst the more weighty are often left behind.

The essay of Mr. Bell, we should have observed, contains some opinions respecting the causes of the circulation of the blood as connected with the state of the fluid itself; but these are so purely hypothetical, that we cannot adduce them amongst

---

(27) There is a very remarkable coincidence between these notions of Mr. Bell, and the mode in which they are expressed, with those advanced in a Thesis by a young candidate for the diploma, Dr. L. C. FEHTZSCH, at Leipzig, a few months since; as will appear from the following abstract, which is given in the Altenburg and Leipzig *Allgemeine Medizinische Annalen*, of January 1819. "Das capillargefässsystem saugt das blut auf, und treibt dasselbe durch eine abwechselnde systole und diastole in die anfange der venen, welche es dann ebenfalls durch eine saugende kraft zum Herzen zurückführen; die arterien treiben das blut durch ihre eigne kraft, welche unter dem einflusse der nerven steht, zurück in das capillargefässsystem, das herz dient dem ganzen kreislaufe zum regulator, wie bei maschinen das schwungrad." We shall conclude the paragraph, though the foregoing is that to which we alluded. "Am herzen, unterscheidet der verfasser, einen dreifachen moment des pulschlages, den man aber nur durch das an den thorax gelegte ohr bemerken kann, der pulsschlag an der hand geschieht gleichzeitig mit dem mittelsten momente des herzschlages."

(28) See his *Recherches Anatomiques sur la Position des Glandes, et sur leur Action.* Paris, 1752.

matters that we consider to be conducive to the progress of science. Indeed, it is with regret that we witness such speculations advanced under such authority, and for the reasons that made one of the most acute and profound observers of the motives of human actions refrain from adducing his own opinion, or any decisive judgment, on an important subject, respecting which nothing more than conjectures could be rationally formed: "Quin etiam obest plerumque iis, qui discere volunt auctoritas eorum qui se docere profitentur, desinunt enim suum judicium adhibere: id habent ratum, quod ab eo quem probant judicatum vident." (CICERO, *de Natura Deorum.*)

Some very interesting facts relative to the subject which has just engaged our attention are related by Mr. JAMES, of Hereford, in the last volume of this Journal; (29) on which Dr. WILSON PHILIP remarked, that he had never seen nor read of a case in which the influence of the nervous system on the blood-vessels, independently of any action of the former on the heart, was more strikingly displayed: and it is one instance of the independance of the action of the arteries on that of the heart under certain circumstances. There are several other important indications in these facts, which, not immediately relating to the present question, we pass over unnoticed.

A recent, and totally unexpected, accident has prevented our receiving the principal part of the medical works published in Italy during the period comprised in this sketch, the want of which we shall supply in the ensuing one. Amongst those which we have received is an interesting little pamphlet, "*Ozii Medici*," in reply to the remarks of an eminent physician of a neighbouring nation, on the physiological doctrines of that country; but we defer the consideration of this for another occasion, when we shall give an extensive view, conjoined with our own observations, of the subject which it involves.

Some ingenious speculations, which may be considered as a development of some of the most important physiological principles of WHYTT, HUNTER, GALLINI, (30) MALACARNE, (31) and BICHAT, have been advanced by Dr. SURUN respecting the function of menstruation, in a memoir presented to the Societé Medicale of Paris. (32)

This physician first advances as a principle, that the same laws of vitality are applicable to the uterus as to the organs in general; and that it does not, as some physiologists have pre-

(29) *Medical and Physical Journal*, vol. xli. p. 130.

(30) *Saggio d'Osservazioni concernenti i Progressi della Fisica del Corpo Umano.* 1792.

(31) *I Sistemi.* Torino, 1800.

(32) *Théorie de la Menstruation, fondée sur les Caractères naturels de la Vie des Organes, et particulièrement de l'Action Nerveuse.* 8vo. pp. 56. Paris, 1819

tended, possess an existence *sui generis*, distinct from the general life of the body. Taking a general view of the animal economy, he thinks it appears evident that there are two species of nervous influence: one, which he terms *organic nervous action*, which regulates only the functions of an organ; the other, the *general nervous action*, which relates to the life of almost all the different tissues. A part, he considers, may be temporarily deprived of the former, (that is to say, disqualified to perform its proper functions,) without losing the second, which relates to the circulation, calorification, nutrition, exhalation, and absorption. These types of nervous influence are again subdivided into *encephalic* or *cerebral nervous action*, *ganglionic*, and *combined*, according as the organ, which evinces it, receives nerves from the brain, the great sympathetic nerve, or from both.

After some corollaries, tending to illustrate these principles, relative to the common actions and proper functions of the stomach and intestines, he shews in what manner they are applicable to the uterus. The ganglionic nervous action serves him to explain all the local or sympathetic phenomena, of which this organ is the primary source from the instant when it enters on its proper functions.

Before the epoch of puberty, he says, we see the uterus animated only by *general nervous influence*, the only species that is then necessary for its nutrition ; but at that epoch *organic sensibility* is developed in it, it becomes adapted for the performance of the function for which it is designed by nature ; and it is towards this object that from that time all the spontaneous, and more or less regular, movements which agitate it, tend.

Dr. Surun after this enters into a more extensive digression, in order to adduce illustrations of these principles from the proper functions and common actions of the lungs, the heart, and the gastric system. Amongst the interesting, and for the most part well founded, reflections which he advances on this occasion, we find the following, which indicates an apparent truth that perhaps is not generally understood. The uterus, he says, enters into action previously to the arrival of its natural excitent, which is the product of conception, just as the heart dilates and contracts independently of the presence of blood in its cavities, the respiratory organs before the introduction of air, and the stomach and intestines before the reception of food.

The truth of the observation relative to the uterus, seems to be shown by the formation of the epichorion in cases of extra-uterine conception. With respect to that on respiration, it seems probable that the state of the bronchiæ themselves has been too little attended to : they have been considered passive, and the influx of air into them entirely dependant on the dilatation of the thorax. The truth of this description seems more than doubtful. May not a species of asthma, unaccompanied by either pain, cough, or subsequent expectoration; coming on suddenly from terror, great mental anxiety, &c. and often disappearing as suddenly on any forcible emotion, depend on the want of *active dilatation* of the bronchial tubes from diversion of nervous influence? It should be observed, that in these cases there is no want of the proper dilatation of the thorax. The notions of Bordeu respecting the action of the glands are very unjustly neglected by the generality of physiologists.

Returning to the interesting memoir of Dr. Surun, we commence with the second part, in which he examines the phenomena that accompany the exercise of the organic nervous action in the uterus, at all times, except during pregnancy. Menstruation he attributes to an habitual erectile motion of the uterine tissue, comparable to the turgescence of the external organs of generation; and which, arriving at its maximum towards the epoch of menstruation, precedes and provokes the arrival of the blood in the small vessels, where it accumulates until the instant of its evacuation by means of the exhalents. This *orgasm*, which he terms the *menstrual erethism*, is considered the only important circumstance for all the purposes of nature. He attributes the accidents which commonly accompany the suppression of the menses, to an alteration of organic sensibility and action, rather than to the want of the flow of blood: this want being properly the effect, and not the cause, of that derangement; and this want of organic sensibility is often only one of the manifestations of a more general disorder of the organic sensibility. In support of this notion, Dr. Surun adduces numerous facts that seem to prove that the actual flow of blood is only of secondary importance: for instance, there are whole nations where women are not subject to it; and in Europe, they often conceive without having ever experienced it. Young girls, too, arrived at an age approaching that of puberty, have become pregnant without having ever menstruated.

The author terminates this part of his memoir with some reflections on the natural relations between the uterus and other parts of the system, and on some accidental anomalies in menstruation. Without attempting to explain the cause of the change which takes place in this organ at the age of puberty, (since our anatomical knowledge of the two nervous systems is not yet sufficiently accurate,) he attributes chiefly to the revolution which is thus effected, for the development of organic nervous influence in the uterus, the extreme susceptibility of women to various sympathetic affections, during the whole period of their fecundity.

The final cessation of the menses, an event so important in itself, and frequently so much to be feared from the trouble it induces in the system, particularly in persons submitted to moral or physical disadvantages, is the last subject of Dr. Surun's reflections. He thinks that women who have lived according to the ordination of nature, have occasion to congratulate themselves on, rather than to lament, the occurrence of this event; since, by it, nature reserves in favour of the individual, now weakened by age, the increase of vitality which had previously been devoted to the re-production of the species.

We have indulged more at length in the consideration of the memoir of Dr. Surun, than at first view might appear appropriate to so particular a subject, according to the plan of this sketch; but we have done so from a conviction that the principles inculcated in it may be applied to various other functions, and that they merit the most profound meditation.

There is one point in this memoir respecting which we are yet disposed to adduce some observations. It is evident that Dr. Surun considers the menstrual evacuation as hæmorrhage, not as a secretion: we believe that this yet remains a disputed point with us. The argument most dwelt upon in favour of the menses being a secretion, in the ordinary sense of that term, is, that the fluid evacuated does not coagulate. After many very accurate and well-conducted experiments (33) with this fluid, Dr. F. LAVAGNA was led to conclude, that it differs from pure blood only in the want of the fibrous part of its composition. This, he thinks, may be attributed, without impropriety, to the fineness of the vessels through which it passes; and that, during pregnancy, those vessels becoming enlarged, permit the pure blood to escape.

The following observations may also be worthy of attention. He found that "the blood collected from the vessels of the funis umbilicalis, which related immediately to the placenta, formed a tenacious coagulum, and contained a considerable proportion of the fibrous substance, though it was a little more soft and gelatinous than that collected from the blood of an adult in the state of health.

"The blood contained in the vessels of the funis which related to the fœtus, hardly coagulated in the smallest degree, and only furnished a very small proportion of fibrous matter." He thence concludes,

"That the gravid uterus, from the various circumstances by which it is then influenced, acquires the power of furnishing blood provided with fibrous matter.

---

(33) *Esperienze sopra il Sangue Menstruo; di* FRANCESCO LAVAGNA, giuniore, Dottore in Medicina, Membro della Societa Italiana, &c. Annali Universali di Medicina, di Milano. No. 17.

"That the blood, in passing from the uterus to the placenta, and thence by the umbilical veins to the fœtus, is abundantly supplied with fibrous matter, which is subtracted from it by the embryo to be appropriated to its own use, for the means of its growth."

An ingenious writer in the Journal Universel des Sciences Medicales, (34) has advanced some objections to the opinions of Dr. BROUSSAIS respecting the primary cause of respiration. (35) He says,

"Let us for an instant admit the existence of this internal sense, (that arising from the want of air, suffered by the internal membrane of the lungs,) we cannot suppose it acting by itself, and without extraneous excitation on the sensitive centre; since, if it did, it would produce the efforts for inspiration long before the birth."

This objection is not valid; since the want was not experienced by the infant whilst in the uterus, and therefore the sensation supposed to be afterwards induced by that want could not then be felt. The following is more solid:

"It remains to be admitted, as M. Broussais has done in his hypothesis, that the want of oxygenation is only experienced on the mucous surface of the lungs; or, if it be required, that the irritation of the skin from the contact of the air, is sympathetically transmitted to the pulmonary mucous membrane, which in turn rouses the sensitive centre."

Any reply to this can only be hypothetical: although expressly applying to the latter proposition, DARWIN's observations and reflections on *concatenated actions*, and those of BORDEU and BICHAT on *the mucous tissue*, will show that a conclusion in the affirmative would be pretty well supported.

This physiologist again says,

"But, without considering that such an exclusion, or such a management, seems but little necessary, and should not be admitted but after the most complete proofs; if the want of respiration were only transmitted to the brain by the lungs through the medium of branches of the eighth pair of nerves, as M. Broussais believes, the division of the two nervous trunks by which it is effected, should always produce instant asphyxia. Now, the experiments of M. Dupuytren, of Legallois, and of several others, prove that such a division of the nerves does not prevent the respiratory phenomena being still prolonged for a considerable length of time; and the animal, far from having lost the sense of the want of respiration, makes violent efforts to dilate the chest."

---

(34) Tome xiii. p. 257.

(35) See *London Medical and Physical Journal*, vol. xli. p. 21.

Some others of the experiments of Legallois, consonantly with those of Dr. Wilson Phillip, shew that when the functions of extra-uterine life are established, a sympathy, or consensus, has taken place between some parts of the different systems of nerves, by means of which one will, to a certain degree and for a certain length of time, support the functions which appear to depend on the other. This sympathy, it is rational to suppose, cannot have existed in the fœtus. The experiments above alluded to seem to shew, that the fifth pair of nerves will thus keep up respiration. (36)

The antagonist of M. Broussais notices the above explanation, but his objection to it is totally inapplicable.

We must adduce the following extract, and with that conclude. The importance of the subject they involve will be a sufficient apology for this indulgence in the notice of what are mere speculations, though of a philosophic character.

" Dr. Broussais has not perhaps sufficiently developed the idea of personal identity *(moi)*, sometimes present and confounded with the sensitive centre; (37) and sometimes absent, and leaving the latter to the sole influence of the viscera. If we might venture to supply what he has not thought it necessary to add, we should say: The sensitive centre is constantly excited to action by the internal sense of the wants which it should contribute to satisfy; it is, besides, excited in the state of watching, by the impressions which arrive at it by the five senses, and by those which result from the combinations of ideas operated in the brain. During sleep, solely excited by the constant wants of the organization, it obeys those alone, and only determines the movements which they require. Let the person awake, the senses carry to the sensorium new motives, which provoke new movements. If, amongst these motives, some one oppose itself to the execution of the movements necessary for the support of life, he can retard it; but the sentiment of want, increasing with the denial to supply its demands, soon becomes superior to the motive which retains the encephalic centre, and this then ordinarily accedes to the imperious sentiment. We would say, *ordinarily*, because there are examples of men, endued with great force of will, incited by a powerful motive, (as the idea of rescuing themselves from tyranny,) who resisted the call for respiration, so as voluntarily to asphyxiate themselves; and, although such effort should have not been always sustained to the point necessary to produce death, the same reasoning would not be less correct, since the want of respiration would have terminated by inducing it.

(36) See *Medical and Physical Journal*, vol. xl. pp. 497 and 8; and vol. xli. pp. 22 and 3.

(37) See *Medical and Physical Journal*, vol. xl. p. 24 *et seq.*

"This applies to the states of sleep and watching. The apoplectic appears to us, in this respect, exactly comparable to a person asleep. In this morbid state, the encephalic centre has ceased, as during sleep, to communicate with the external senses; but it continues to receive the impression which results from the internal want of respiration, and it still induces the respiratory movements. The difference which exists between a person awake and one in the state of sleep or of apoplexy, consists then in this, that, in the former, the encephalic centre communicates with the external organs of the senses, and gives to the individual the consciousness of impressions external as well as internal; whilst, in the other, he only receives the impressions of the internal senses, of which the individual does not appear even to take notice. It is that, we think, which M. Broussais signifies by the presence and the absence of the perception of self-identity (*moi*)."

We might say, in borrowing the language of Bichât, that the encephalic centre appertains both to organic and to animal life; that, as relates to the first, it is constantly in action; whilst its function with respect to the second is intermittent, like that of the other organs of relative (animal) life. It is as an organ of animal life that the sensitive centre constitutes the *moi*.

Another of those cases which contribute to involve the function of generation in inexplicable mysteries, has recently occurred to the observation of Dr. CHAMPION, of Bar-le-Duc. (38) On visiting a woman who had been three days in her first labour, he found the orifice of the vagina obstructed by a firm and very thick membrane, except in two points, so small that the largest of them would hardly admit a stilette of the dimensions of a pin's head. The urethra was so much dilated as freely to admit the fore-finger to pass into the bladder. On dividing the obstructing membrane, the vagina was found of the ordinary dimensions. The woman produced, without any remarkable circumstance occurring, twins of the two sexes.

The following is the previous history of the case, in the expressions of Dr. Champion:

"At twelve years of age, Mary R*** experienced pains in the loins, which continued three days, and returned every three weeks. After the lapse of some months, a tumor appeared in the right iliac region, which sensibly increased in size, particularly when the pains in the loins came on. With these pains, a difficulty in passing the urine was sometimes conjoined. Her parents thought she had stone in the bladder: surgeons were consulted, who sounded her without finding any; nor did they discover the membrane obstructing the vagina.

(38) *Bulletin de la Société Medicale d'Emulation*, Mai 1819. Journal Universel des Sciences Medicales; No. 41.

"Between the thirteenth and fourteenth years of her age, the menses appeared after the third day of the periodical pains in the loins. The blood was very black and fetid; it flowed in filaments, and continued during eight days, but only when the patient was in the erect or sitting posture.

"The tumor of the abdomen disappeared immediately after this evacuation, and never afterwards returned. The menses occurred regularly every third week, flowing drop by drop, and during three days; the first of which, the pains in the loins were so severe that the patient was obliged to keep in bed. This dysmenorrhœa has not re-appeared since her accouchement.

"With respect to the epoch at which the coitus took place in the urethra, I could not exactly ascertain it; but every thing leads to a belief that it occurred much subsequently to conception, because her accouchement happened nine months and fifteen days after her marriage. The severe pain which the woman experienced in the first cohabitations, induced her to separate herself from her husband, who then formed such suspicions respecting the motives for this conduct, that there remained no way to remove them but to give up herself to his discretion. She then suffered severely from his approaches; and it was not until towards the fifth month of her pregnancy, that he could introduce the penis deeply into the urethra."

This case appears to support very strongly the opinion of those physiologists who think that, the influence of the semen virilis on the ova is effected through the medium of the system.

We shall only give a reference to a little work (39) by M. Bourdon, containing some observations tending to prove the necessity of active contraction of the stomach in order to effect vomiting. As the opinion on this function revived by Dr. Magendie, and, as he considered, substantiated by experiments, was never adopted amongst us, it will be unnecessary to produce arguments against it: besides which, we have already, perhaps, sufficiently considered this subject. (40)

A work on the physiology, &c. of the teeth, (41) by Dr. Delabarre, must not be passed over unnoticed, although we cannot give a particular account of its merits. This is the first of a series of volumes that will hereafter appear, and is occupied

---

(39) *Memoire sur le Vomissement*, par Isidore Bourdon. 8vo. pp. 55. Paris, 1819.

(40) *Medical and Physical Journal*, vol. xl. p. 532.

(41) *Traité de la seconde Dentition, et Méthode naturelle de la Diriger; suivis d'un aperçu de seméiotique buccale; orné de 22 planches.* Par C. F. Delabarre, Docteur en Medecine de la Faculté de Paris; Chirurgien-Dentiste du Roi; Professeur de Maladies de la Bouche a l'Administration générale des Hôpitaux civils de Paris. 8vo. pp. 311. Chez Mequignon-Marvis et Gabon, Paris; et Treuttel et Würtz, London.

chiefly by physiological and pathological remarks, &c. with the therapeutical measures that come within the usage of the general practitioner. The following ones will treat of that part of the subject which constitutes expressly the art of the dentist. Those who desire to acquire a thorough knowledge of these matters will, doubtless, from the known talents of the author, find it a very valuable possession. Of the contents of the first volume we can speak with decision, that it well evinces what the author indicates in the following paragraph:

"Le médecin qui, après avoir acquis les connaissances générales de son art, se livre spécialement à l'étude d'une seule branche, s'aperçoit bientôt qu'elle est susceptible d'une grande extension."

Before we quit the subject of physiology, we should notice, as one of the productions of the period conducive to the improvement of medicine, the memoir of Dr. BACHE, of Philadelphia, on the effects of temperature on the animal economy; but it must only be to give a reference to it. (42) Any thing like a particular examination of it would extend to a length unappropriate to the limits of the present essay, especially as they must be mere speculations.

§ III. Hitherto we have treated of the physical construction of the human body, and the manifestations of its functions, in the state of health alone: let us now consider the peculiar phenomena it evinces when suffering from some of the various forms of disease. But, it will be proper that we should in the first instance say, what is to be regarded as disease: this we shall do in the expressions of a physician, whose genius was expressly adapted for the philosophy of medicine.

"By disease, should be understood a derangement in the functions, dependant on some organic vice, or augmented or diminished action of some part; for, we are sick, it has been said, when our functions are troubled; or when the energy, the tone, of our organs, is destroyed. We may find in the works of Aretæus, and some other physicians, traces of this *organism*, which has lately been so much better comprehended and developed than at any former period. As it is on this organism, well understood, that the knowledge of health and of diseases depends, it will be useful to connect with it the observations which we shall hereafter relate. We require, for the existence of health, a regular and determinate order and connexion in the organic movements: when they deviate from this harmony, what is termed indisposition or disease is the result." (43)

(42) *London Medical and Physical Journal*, vol. xli. p. 259.

(43) BORDEU, *Réchèrches sur les Maladies Chroniques.* Théor. xvii. Paris, 1775.

This paragraph, remarkable when the period at which it was written is considered, describes with precision what is now becoming generally admitted as the first principle in pathology. This doctrine is universally adopted in France; it has been for several years progressively acquiring perfection in Italy, by the labours of MALACARNE, GALLINI, RASORI, FRANCESCHI, and TOMMASINI; and, since the works of HUNTER have been duly reflected on, it has gradually advanced in our own country. It forms the basis of the system of DARWIN: and here we cannot avoid remarking, that the *Zoonomia* seems to be too much neglected by us. We know that many prudent and judicious physicians are disposed to caution students against applying to it, rather than to recommend it to their attention, fearing its undue influence at a period of life, when the understanding might be impressed with an idea that its apparent beauty and simplicity were the traits of nature: but, when the mind is cooled by due reflection, and well informed by practical experience, we think that there are but few works of equal extent, from which physicians in general may derive more important ideas for their own meditation. Before we turn from this slight and partial view of the medical doctrines now prevalent, we should observe, that the same principle is almost established in Germany. Many judicious physicians in that country have for several years combatted the pure Brunonian hypothesis, by their practical illustrations of its errors; and several excellent physiologists are, at the present period, theoretically completing its destruction. (44)

We recur to our professed object.

We shall first notice the systems of pathology which have been published, and then devote our attention, expressly, to particular treatises and individual observations.

Of the work of Mr. ALLAN on *pathological and operative surgery*, (45) viewing it generally, we should speak in terms of the

---

(44) The work of Dr. SCHAFFER, bearing the ensuing title, will be perused with much pleasure and advantage; as, besides much valuable practical matter, it will be interesting by showing the mode in which some of the principles of KANT are applied to physiology by his compatriots. "*Versuch eines Vereins der Theorie und Praxis in der Heilkunst;* von Dr. Joh. Ulrich. Gottlieb Schaffer; furstl. ott. Wallerst. Hofrath u. Leibarzt, ehemahl. furst. primat. Sanitatsrath, des Instituts der Moral und schonen Wissenschaft u. der Physicalisch-Medicinischen Societat zu Erlangen Mitglied. Berlin, 1818." The following brief propositions, taken from it, comprise a specimen of what we have above alluded to: "Der ganzen korper durchdringende form und mischung belebter materie, find das oberste princip der organisation, des lebens." "Der individuelle organismus ist abdruck, refler desuniversums. Das leben jenes ist daher dem letztern nur abgeborgt."

(45) *A System of Pathological and Operative Surgery, founded on Anatomy; illustrated by drawings of diseased structure and plans of operations.* By ROBERT ALLAN, Fellow of the Royal Colleges of Surgeons, of London, and Edinburgh; Surgeon to the Royal Public Dispensary for the city and county of Edinburgh; and Lecturer on Surgery. 3 vols. 8vo. Edinburgh. Thomson and Co.

warmest approbation. As a system, it is not to be expected that there should be found in it much of original novelty on any particular subject: indeed, this would not be adapted to the chief object of works of this kind, which should be to comprise a lucid and correct exposition of the principles most generally admitted by judicious practitioners. This is well effected by Mr. Allan.

When treating of venereal bubo, he however advocates an opinion by no means generally adopted, but which we were pleased to find made a subject of disputation. It is, that the inflammation of the gland depends merely on the transmission of irritation from the sore, not on absorption of virus. Yet, when speaking of cancer, he says, "Whilst all this destructive process (ulceration) is going on, the disease sooner or later extends to the axillary glands, by the inflammation spreading along the lymphatics, *or by absorption.*" Mr. Allan has not expressed any particular opinion respecting the point to which we have alluded, on this occasion; nor has he considered it in a general manner.

We probably attribute to the absorbant vessels, functions which they do not perform. M. Larrey is of opinion that they only transmit such matters as are appropriate for the nutrition of the body, and the lymph generally effused by the exhalents. He thinks that even simple pus is not taken up by them; and says, that in all the bodies he has examined of men who died while suffering under extensive suppurating wounds, abscesses, &c. he never once detected that fluid in any part of their course. He considers it is the veins which pass into the general system, the various irritating substances which have been supposed to enter it by the absorbents. The experiments of Mr. Brodie with poisons applied to wounds in the extremities of brute animals, powerfully support this opinion. Here it was certain that the destructive matters did not enter the system by the absorbents, and that they did pass into it by the veins. The transmission of the oxide of mercury, and some other substances, by the absorbents, should not, as might be done on a slight consideration of the subject, be considered an objection to the above notion; since it is evident that such substances as do not add to the irritative properties of the lymph, chyle, &c. may be passed with those fluids; whilst those whose stimulative powers are above the due relation to the sensibility of the vessels, would be arrested or rejected by them. The action of various medicines, received into the stomach, on distant parts of the system expressly, is not a more valid objection than the foregoing one; since substances which are not excitant with respect to the absorbents of the intestines, &c. may be so to the capillary vessels of another part of the economy, to whose sensibility they bear a different degree of relation.

Although this notion is now merely hypothetical, there are such arguments in favour of it, in both positive and negative evidences, as shew it to be worthy of attentive consideration.

The *Elements of Pathology* (46) of Dr. CAILLIOT, is a work possessing a considerable degree of merit; though it contains but few propositions of striking novelty, when considered individually.

We shall give a general view of the arrangement of the work, and cite two or three paragraphs which are distinctly interesting.

The first section is devoted to general reflections on life, the vital properties, sensibility, contractility, sympathies, the epigastric centre, animal heat, the cellular tissue, the vertical and horizontal divisions of the human body, and the fluids in general.

In the second part, he treats of diseases in general; of their seat, nature, and differences.

In the third, of etiology.

The fourth is devoted to what relates to the course and termination of diseases; their periods, crises, conversions, metastases; to convalescence; and to the alterations which take place, in their progress, in the structure of organs.

In the next place, he considers what he calls *the sensible phenomena of the derangements in the properties of parts and their functions:* in plain terms, the *symptoms* and *signs* of diseases.

What relates to therapeutics terminates the work.

This arrangement is lucid, and better adapted for elementary instruction than that in which each group of symptoms, termed a disease, is separately considered; since, on the plan of Dr. Cailliot, there are so many opportunities for adducing remarks on different morbid affections, illustrative of their relations, in a concise and perspicuous manner.

We like his mode of describing the particular vitality of each organ. The *vital principle*, he says, "is not exclusively placed in any particular part; it vivifies and animates the whole system; it is modified in such a manner with organization, that, as this varies, the same parts seem penetrated with different properties."

He thinks, "the animal economy appears to have but one 'dose' of sensibility; and consequently, this cannot be concentrated in one point without being diminished in another."

This admits of dispute. In hysterical women, for instance, the voluntary muscles possess great increase of their ordinary power, whilst no function appears to be relatively diminished.

---

(46) *Elémens de Pathologie générale et de Physiologie Pathologique;* par L. CAILLIOT, M.D. ancien Médecin en chef des Armées Navales et de la Marine, &c. 2 tomes. 8vo. Caille et Ravier, Paris. 1819.

This is an interesting question in its application to medicine; particularly as regards the use of counter-stimulants, now becoming such important agents in the hands of the enlightened practitioner. It must be observed, that many distinguished physiologists admit the truth of the proposition adopted by Dr. Cailliot; and it is not easy to adduce, from reasoning alone, any arguments by which it can be successfully combatted.

The following remarks on the principal traits which distinguish chronic and acute diseases, are deduced from accurate observation:

"The energy of vital re-action which takes place in acute diseases, forms their fundamental character. They are not only acute by the rapidity of their progress, and the promptitude of their termination; they are so likewise by their intensity, and the evidence of the phenomena by which they are characterized. They do not less differ from chronic diseases by their effects on the system, than by the duration of their course. They often affect the vital properties without injuring the structure of the organs, which is only secondarily affected; whilst the proper character of chronic diseases is to produce a sensible alteration of their substance, without the vital properties appearing to be deranged, they only seem to become so secondarily."

The elementary treatise on pathology (47) by Dr. Bartels, of Breslau, should not be passed totally unnoticed, although we shall not adduce any observations from it: as, being intended principally for his class of pupils, it is not calculated to excite much general interest. But, as it is concise, and contains much interesting matter judiciously arranged, we would recommend it to medical students in general, who understand the German language. We may here observe, since it is becoming not an unusual thing for English students to visit the schools on the continent, that they will find the perusal of such works of great utility to them, previously to their journey; tending to make them well acquainted with medical expressions, &c.

The various works on *fever*, that, with respect to the period of their publication, come within our view, will now engage our attention; but, in a general manner only; since the most important of them will become subjects of regular "Critical Analyses." Treatises on this subject have been produced by Drs. Cheyne, (48) Percival, (49) Clutter-

---

(47) *Lehrbuch der Allgemeinen Pathologie;* von Dr. Ernst Bartels, ordentl. Prof. der Medizin an der Konigl. Universität zu Breslau, &c. &c. 8vo. viii. und 207 s. Breslau, 1819.

(48) *Report of the Hardwicke Fever-Hospital;* by John Cheyne, M.D. F.R.S.E. M.R.I.A. &c. In the *Dublin Hospital Reports,* vol. ii. Hodges and M'Arthur, Dublin; and Longman and Co. London.

(49) *Practical Observations on the Treatment, Pathology, and Prevention, of Typhous Fever.* By Edward Percival, M.B. M.R.I.A. &c. London. 8vo. pp. 156.

BUCK, (50) ARMSTRONG, (51) ROGAN, (52) WELSH, (53) MILLS, (54) PROUDFOOT, (55) DICKENSON, (56) HALE, (57) HUFELAND, (58) MILIUS, (59) and LASSIS. (60)

The Report of Dr. Cheyne will become a subject of particular consideration on a future occasion. This excellent specimen of accurate clinical observation and judicious induction, will therefore be adverted to in the present sketch, only for the purpose of illustrating any points respecting the pathology or treatment of fever, which may be selected in the course of this history as objects for especial remark.

The work of Dr. Percival has a decided title to the praise due to eminent merit; but, as this treatise is a development of his opinions respecting the epidemic fever of Dublin which were published in the first volume of the Hospital Reports of that city, and were noticed in the review of that volume, it will not be necessary for us to designate in this place the character of the doctrine it contains.

Dr. Clutterbuck continues to advocate the doctrines he ad-

---

(50) *Observations on the Prevention and Treatment of the Epidemic Fever at present prevailing in the Metropolis.* By H. CLUTTERBUCK, M.D. 8vo. pp. 299. London.

(51) *Facts and Observations relative to the Fever commonly called Puerperal.* By J. ARMSTRONG, M.D. 2d edit. 8vo. pp. 240. London, Baldwin and Co.

(52) *Observations on the Condition of the Middle and Lower Classes in the North of Ireland; and on the late Epidemic Disorder, as it prevailed in an extensive District of that Country.* By FRANCIS ROGAN, M.D. Physician to the Strabane Fever-Hospital, &c. 8vo. pp. 159. Whitmore and Fenn, London.

(53) *A Practical Treatise on the Efficacy of Blood-letting in the Epidemic Fever of Edinburgh, illustrated by numerous Cases and Tables, extracted from the Journals of the Queensbury-House Fever-Hospital.* By BENJAMIN WELSH, M.D. Superintendent of that Institution, and Member of the Royal Medical Society of Edinburgh. 8vo. pp. 150. Bell and Bradfute, Edinburgh; and Longman and Co. London.

(54) *The Morbid Anatomy of the Brain in Typhous Fever; to which are added, Cases of the present Epidemic, with Remarks on its Nature and mode of Treatment.* By THOMAS MILLS, M.D. &c. 12mo. pp. 67. Hodges and M'Arthur, Dublin; and Underwood, London.

(55) *Account of the Endemic Fever of Spain, as it occurred at Carthagena in 1812.* By THOMAS PROUDFOOT, then Assistant-Surgeon, 27th Regt. In the *Dublin Hospital Reports*, vol. ii. Hodges and M'Arthur, Dublin; and Longman and Co. London.

(56) *Observations on the Inflammatory Endemic incidental to Strangers in the West-Indies from temperate Climates, commonly called Yellow Fever.* By NODES DICKENSON, Staff-Surgeon to his Majesty's Forces, &c. 8vo. pp. 216. Callow, London.

(57) *History and Description of an Epidemic Fever, commonly called Spotted Fever, as it appeared at Gardiner in the United States.* By E. HALE, M.D. M.M. S.S. 8vo. pp. 246. Wells and Lilly, Boston; and Souter, London.

(58) *Practisches Handbuch der Heilkunde der Fieber und Entzundungen.* Von C. W. HUFELAND, M.D. Königl. Preuss. Staatsrath. Prof. der Medicin auf. der Universitat zu Berlin, &c. &c. 8vo. Jena.

(59) *Waarneming feener Zenuwachtige ontstekingskoorts, benevens eenige algemeene Aanmerkingen over de Geneeswyze der Zenuwkoosten.* Door P. J. MEESTERS MILIUS, M.D. en Officier van Gezondheid der Tweed Klasse. 8vo. pp. 260. Abrahams, Middleburg.

(60) *Recherches sur les véritables Causes des Maladies épidémiques appellées Typhus, ou de la non Contagion des Maladies typhoides.* Par M. LASSIS, M.D. de la Faculté de Paris; ancien Médecin des Armées, &c. &c. 8vo. pp. 333. Paris, 1819.

vanced in 1807, that inflammation of the brain is exclusively the primary cause of fever. This doctrine was distinctly advanced, in a less exclusive manner, as early as the year 1785, by Dr. PEW; (61) and was, indeed, pointed out as a frequent cause of it, by Dr. GILCHRIST, of Dumfries, at a much earlier period. (62) We seem to have let some of our best practical writers fall into oblivion in a very extraordinary manner: the treatment of fever adopted at the present period, is not apparently better than that employed by Dr. Gilchrist.

Dr. Clutterbuck says, "Practical illustrations are issuing almost daily from the press, yet it does not appear that we are advancing a single step towards either a consistent theory, or useful practice, in fever." Page 244. This (when we consider the numerous treatises on the subject, by men of the first-rate talents, which have recently appeared, and all the information which anatomy has furnished,) appears at a first view to be a very extraordinary assertion; but, when we recollect the sentiments expressed in the introductory part of the same work, which shew the spirit that gave rise to these remarks, the mystery is dispelled. When speaking of the difference of opinion existing between himself and Dr. BATEMAN, Dr. Clutterbuck says, "There is, however, such an approximation, that I am willing to flatter myself, a further candid consideration of the subject will have the effect of obliterating the slight shades of difference that subsist between us. Whenever this is accomplished, I shall consider I have achieved a triumph."

We include the Treatise on Puerperal Fever by Dr. Armstrong, amongst the important additions to the medical literature of the present period, although a second edition, in consequence of its comprising numerous observations and reflections still further illustrative of the nature of that affection, and of the propriety of the mode of treatment so forcibly inculcated by the author on the first publication of the results of his experience in that disease. This work will engage our particular attention on a future occasion; yet, the interesting nature of the subject induces us now to remark, that it is not excelled in point of apparent accuracy of clinical observation and judicious induction.

The work of Dr. Rogan is principally devoted to observations respecting the origin of the fever at present epidemic in Ireland, the extreme poverty and unparalleled miseries of a great proportion of the people of that country, and to proposals of measures for mitigating some of those evils, and the diseases which thence result: the reflections of Dr. Rogan, on this subject, are as judicious as they are benevolent. This part

(61) *Medical Sketches.* 8vo. London, 1785. (62) *An Essay on Nervous Fevers;* in the Edinburgh *Medical Essays and Observations,* for the year 1736.

of the work is chiefly of local interest only; but, as far as this extends, it should be attentively perused. The remarks of the author relative to the pathology and treatment of the malady, are conformable to the doctrines to which we expressly give our approbation.

The work of Dr. Welsh is chiefly interesting, and more expressly valuable, as a therapeutical dissertation: we shall therefore defer taking a particular view of it until we arrive at the section of this sketch devoted to observations on that part of the application of medical science.

The little tract of Dr. Mills is particularly valuable as an addition to such knowledge as anatomy will furnish respecting the cause of typhous fever. The researches of Dr. Mills have shewn that the brain is an organ very frequently affected with inflammation in that disease, either primarily or secondarily. "In the majority of the patients," says Dr. Mills, "whose cases I have briefly stated, the brain was the organ primarily and principally engaged; but the disorder had sometimes its seat elsewhere,—in the liver, the lungs, or the alimentary canal."

It is with much pleasure that we witness so accurate an observer advocate the doctrine of the essential and primary existence of local inflammation in typhous fever; though we consider he is too restrictive, in not admitting that inflammation of parts will ensue, as a consequence of that previously existing in others, during the course of the disease. He appears to neglect too much the influence of the nerves on the arteries distinctly from affection of the heart, with the consequences of this influence; and the effects of what is usually termed *sympathy*. Such are the ideas we form from his remarks; but, as they are very concise, we shall wholly transcribe them:

"I shall now close what I had to offer, by a few observations on the phrase 'determination of blood,' so often employed by physicians, who attribute the pain, heat, weight, and uneasiness, felt in different organs during fever, to such determination; and, while they allow the application of leeches and blisters for the relief of the affected parts, they regard this as a secondary object, (the affection itself being but secondary, the consequence of febrile actions in the vessels of the system at large,) where fever is seated, or in what it consists, are points on which they declare themselves incompetent to decide.

"May we now ask, can there be a determination of blood to any organ, as a consequence of the febrile actions of the general system?

"The heart and the blood-vessels transmit the blood; each organ is supplied with a regular determinate quantity, proportioned to its wants, the importance of its functions, and the quantity of the circulating mass. Where general plethora exists, there is an increased fullness of the vessels of every part and organ; and the reverse takes place, where there is a deficiency of blood.

"But, where lies the power of transmitting to different organs, at different periods, a preternatural quantity of blood?

"The heart and blood-vessels are the great movers and conduits of the circulating mass.

"Does the power lie here? This is not admitted by any physiologist, nor is it consistent with reason or the laws of the animal economy. How, then, are we to account for the pain, the throbbing, the sense of weight or fullness, felt in fever, in different organs?

"In the same manner and on the same principle as in catarrh, enteritis, phrenitis: in these diseases, local inflammation exists, and the general febrile actions are the consequence of such local inflammation. So it is with fever; and this I have endeavoured to shew, from an examination of its causes and phenomena, from the effects of venesection, and from the appearances exhibited on dissection.

"Thus, fever is essentially the same, depending on local inflammation: it has, indeed, its varieties and modifications. These depend, in a great measure, on the exciting causes; but still more on the patient's temperament of mind and body, and on the diseases with which he was previously affected."

We regret that Dr. Mills has been so concise in his remarks on this subject. It is not evident from them, whether he absolutely denies that local inflammation ever occurs as a consequence of fever, and its local and immediate cause conjoined; or, whether he only considers that it is never a consequence of the general re-action of the system *solely*. If he means the former, we differ from him in opinion: if the latter, we agree. Our reasons have already been detailed.

Mr. Proudfoot's observations relate to a disease that was epidemic amongst the British troops at Carthagena, in 1812, and which was considered to be yellow-fever. The following brief paragraph will give an idea of his opinions respecting its cause and character.

"Yellow-fever has been variously described by different authors, as they considered the disease contagious or non-contagious, continued or remittent. The term itself, as has been often remarked, is very objectionable, and apt to lead the inattentive into serious error: perhaps it would have been better, had that disease been placed in the order phlegmasiæ, and described as an inflammation *sui generis*, excited chiefly by cold applied to the surface of the body; the parts having previously acquired a certain susceptibility to disease, from the state of the mind over the constitution, and from exposure to marsh miasmata under a high atmospheric temperature."

The above observations, though applied to yellow-fever generally, relate also to the disease which was the subject of the present observations; from which we shall transcribe another paragraph, and with that conclude.

"The appearances upon dissection were uniform, and satisfactory, in as far as they agreed with what might have been expected from the symptoms; being chiefly an inflamed state of the stomach and intestines. Upon opening the abdomen and reflecting the omentum, in many cases, nothing of a morbid appearance presented itself; in others, fasciculi of red vessels could be seen running on different parts of the intestines; and when the disease commenced with great violence, and speedily proved fatal, the whole of the peritoneum was much inflamed. Upon making an incision into the stomach, the villous coat appeared crowded with innumerable minute red vessels, forming specks on different parts, of a beautiful scarlet colour; but the appearances varied according to the violence of the inflammation, which in general was greatest at the cardiac orifice. In many cases, the latter part, at first, appeared quite black and mortified; but, upon investigation, this appearance was proved to depend upon an effusion of blood between the nervous and villous coats of the stomach. I have seen the villous coat entirely destroyed in different places, leaving little excavations, and a plexus of innumerable enlarged and denuded blood-vessels; several of these being ruptured."

The treatise of Mr. Dickenson on yellow-fever is too interesting and important to be briefly considered. This valuable work will engage our attention on a future occasion, when it can be applied to in a more appropriate manner.

As the work of Dr. Hale on the spotted-fever of the United States has already been reviewed in this Journal, we need now only record it amongst the valuable additions to the medical literature of the present period.

The practical manual of the treatment of fever and inflammation, of Professor Hufeland, is worthy of the author of the *Macrobiotics;* and it constitutes a valuable addition to his general system of the practice of medicine. The opinions of the author are in general conformable to those now prevalent in Europe; and his practical application of them is judicious, but incapable of being described in an abstract. This physician, we may observe, is one of those who have most contributed to effect the destruction of pure Brunonism, which was at one period so universally prevalent in Germany, and which is yet sufficiently so to counterbalance, probably, all the benefit the practice of the medical art in that country can effect.

Dr. Milius, for his observations on nervous inflammatory affections, and remarks on the treatment of nervous fever, merits a civic crown from his compatriots; for, although his doctrines are not novel, being conformable to those on which we have long conferred our approbation, yet the boldly advancing of such in Holland, where Brunonism (53) in its worst forms, conjoined with an unintelligible humoral pathology, has been so popular and so prevalent, claims no small degree of praise.

The doctrine of the connexion of inflammation with *nervous* fever, Dr. Milius observes, is either not understood in Holland or publicly opposed ; and it is to explain that doctrine, and to overcome that opposition, not from a notion that he can add any thing of much value to the excellent works already extant on the subject, that he is induced to take up his pen.

His remarks, it appears, particularly apply to the opinions advanced by Dr. Kirkhoff, a physician to the garrison hospital at Antwerp, in a work which he published last year ; wherein he states, that nervous, putrid, malignant, and yellow-fever, are of one and the same nature ; should be termed *idiopathic asthenico-nervous fever ;* and treated by the most powerful stimulants. Antiphlogistic measures, especially blood-letting, Dr. Kirkhoff says, are in no cases useful, and almost always destructive.

Dr. Milius, in his work, adds the results of his own experience to the statements of other physicians, to confirm the truth of the doctrine that nervous fever is dependant on local inflammation. He also describes the appropriate mode of treatment, in a lucid and accurate manner.

Dr. Lassis has written with a view to show, that what is ordinarily called typhus is not contagious. We shall, in the first instance, transcribe a paragraph, to show, if possible, the nature of the malady to which he applies the term typhus.

" J'ai seulement en vue ces maladies connues autrefois sous le nom de peste, ou de maladies pestilentielles, et que l'on désigne assez souvent aujourd'hui sous celui de typhus, lesquelles ordinairement, sont epidémiques, accompagnées de stupeur, et se développent dans les hôpitaux, les lazarets, les prisons, les camps, les vaisseaux, les villes assiéges, sur les bords des canaux en Egypte, sur ceux des criques en Amérique ; en un mot,

---

(53) It must not be supposed, because we reprobate *Brunonism*, that we are insensible to the merits of the doctrines of Brown. They are far different in reality from what they have been interpreted to be : there is much of them, indeed, in the present system of *organism*. It is the false and fanatic manner in which they have been applied in practice, against which we exclaim without reserve.

dans tous les lieux où s'élèvent des émanations méphytiques, et surtout dans ceux où les hommes sont réunis en trop grand nombre. Si quelquefois, ce que je dirai se rapport à la maladie généralement encore appellé peste, ce sera parce que j'y serai entrainé par le sujet, et non avec l'intention de decider pour le moment, a l'égard de cette maladie, la question de la contagion."

The want of precision evident in the above paragraph, when accurate description of the subject of disputation was so essential, in order to render the author's arguments of any force, is but a faint specimen of the vagueness of manner, and indeterminate general statement, that pervade the whole of the work. We hardly ever meet with a logical argument; and in his references to, and citations from, other authors, Dr. L. has frequently given a signification to their opinions totally different from that which they bear in the original. Besides which, he has been improperly partial in the selection of his authors: hardly any of the best of the modern writers on the subject are noticed.

Dr. Lassis also endeavours to show, that the ancients did not believe in the dissemination of febrile diseases by contagion: but the contrary is satisfactorily proved, as evinced in the quotations adduced by Dr. Yeats, in the paper inserted in the last volume of this Journal. Dr. Lassis, apparently not acquainted with the Greek language, when referring to the Grecian writers, uses the Latin translations, in which the word *contagio*, or its derivations, is used instead of the more explicit expressions of the original; and he then quotes a remark of Heurnius on that term, "*Nullus tamen veterum scripsit quid illud sit?* This is true; and, did our ideas of what the notions of the ancients were on this subject, depend on that which we could derive from the sense in which the term *contagio*, individually considered, was used, they would not be determinate. Lucretius and Virgil sometimes apparently use it in the sense of *contango;* whilst Cicero employs it, we believe solely, in that of *contingo;* and expressly gives the Greek συμπαθεια, as synonymous with it. He uses it to describe the similarity of form, manners, &c. often observed in brothers; as well as when speaking of the supposed influence of the planets on animals and the events of nations. (64) But, the determinate sense of the passages adduced by Dr. Yeats, is sufficient to prove the contrary to the statement of Dr. Lassis. The work of this physician is, however, not devoid of some sort of merit: much industry and patient research in dull channels, have been employed in the compilation of it. The "*notices historiques*" of, probably,

---

(64) See *De Divinatione*, lib. ii, *passim;* and *De Fato*, near the commencement of the book.

nearly all the most severe and destructive epidemic maladies on record, will be of very considerable value to other writers, who may hereafter treat on the subject in an historical manner.

Although the decision of the dispute respecting the dissemination of various forms of febrile disease by contagion, is not of much consequence as regards pathology, it is of the first importance in its relation to hygiene. The evils that necessarily result from a refusal to admit such an agency if it exist, have been sufficiently adverted to; but those which must also arise from the attributing of contagious properties to such as do not possess them, although hardly inferior, have not in general been equally reflected on. An excellent memoir on this subject has been lately published by Dr. Potter, of Maryland, (65) which was originally delivered before the convention of the Medical and Surgical Faculty of that state. The author has taken an extensive view of the epidemics that have appeared since 1741, and which have been termed yellow-fever by the best writers on the subject; and introduced some very judicious remarks, deduced from extensive erudition, on the doctrine of contagion, in its general application. He has very powerfully supported (we think contributed to confirm) the opinion that the inflammatory endemic of hot climates, ordinarily termed yellow-fever, is not essentially contagious. He admits that the disease may be communicated by the air surrounding a number of patients of that disease, closely confined under certain circumstances; for instances, in the vessels the General Greene, the Hankey, and the Busbridge; but he observes, "This local infection of the atmosphere is not contagion: those who have thence deduced arguments in support of contagion, have mistaken the person for the place." "The annals of the world cannot furnish an instance of the multiplication of the yellow-fever, from effluvia proceeding from a vessel." The term contagious, as applied to a morbific agent, Dr. Potter considers should be used only to designate a peculiar and determinate secretion of the human body; whilst atmospheric infection may be the result of various putrefying animal and vegetable substances; and it is the latter which always give rise to yellow-fever.

In our last historical sketch, we adduced some observations relative to *cow-pox*, which were apparently calculated to destroy, in some degree, the full confidence which had been generally entertained in its prophylactic powers against variola: our report on this subject on the present occasion, will be more gratifying; as, nothing further has occurred adverse to its best

(65) *A Memoir on Contagion, more especially as it respects the Yellow Fever.* By Nathaniel Potter, M.D. Professor of the Theory and Practice of Medicine in the University of Maryland, &c. 8vo. pp. 117. Coale, Baltimore; and Souter, London. 1818.

interests; and we have several traits to add to the history of its benefits to society.

Of the observations which have since then been published respecting it, we have already given those of Mr. Brown, (66) Dr. Thomson, (67) Dr. Pew, (68) and Dr. Bent: (69) we shall now complete the history.

A committee was instituted at Marseilles in the latter part of the last year, for the purpose of an inquiry respecting the nature of the disease that had been prevalent in several of the departments of France, and which was reported to be small-pox occurring after vaccination. (70)

The first action of the committee was to inoculate four children, who had not been vaccinated, with matter taken from a patient (a girl thirteen years of age, who had cow-pox at an early period after birth,) having the epidemic disease; and which the attending physician believed to be variola. In point of time, of appearance, and duration, the disease produced in the arms somewhat resembled that from inoculation with cow-pox; but it had no determinate character. It was in these cases only local, though accompanied with some degree of fever.

Two children who had been vaccinated, were also inoculated. In one, an inflammatory redness ensued round the wound: on the fourth day, fever appeared, and an eruption of pustules around the point of insertion of the matter, each having at its base a very extensive tubercle, and being terminated at the apex by a vesicle filled with purulent matter: on this a yellowish crust afterwards formed, from under which puriform matter continued to ooze.

In one child, who had not had cow-pox, the pustules on the arm attained a considerable size; and on the ninth day had become confluent, forming a large yellowish crust, which occupied the exterior and anterior surface of the arm: on the twelfth day, pustules appeared over the whole surface of the body, presenting in their development and progress the same appearances as those on the arm. This child had fever, uneasiness, restlessness, vomitings, and pain in the bowels. The arm at length became covered with a gangrenous eschar, followed by an ulcer of an ill character, which terminated by giving rise to an adherent cicatrix.

The whole of the children were of ages between two and eight months.

---

(66) *London Medical and Physical Journal*, vol. xli. p. 271.

(67) *Ibid.* p. 94. (68) *Ibid.* p. 288. (69) *Ibid.* p. 457.

(70) *Rapport du Comité du Dépôt de Vaccin, séant à l'Hôtel-Dieu de Marseille, au Préfet du Department des Bouches-du-Rhône; signé par* Delacourt, *vice-président;* Moulaud, *vaccinateur en chef;* Dugas, Roubaud, Seux, Serrier, Segaud, Cauviere; *et* Robert, *secrétaire-archiviste.*

The negative and indeterminate results of these experiments prevented the repetition of similar measures.

The eruptions in those who received the disease apparently by infection, appeared in various and irregular forms, having sometimes the appearance of a pemphygoid vesicle, sometimes that of a dartrous pustule; sometimes in small, hard, red, tubercles, pointed at the apex; and occasionally bearing some resemblance to that of variola. Although children were chiefly affected, many adults, some of whom had taken the small-pox by infection, were also subject to it. It was supposed by the committee, that small-pox, and varicella in various forms, appeared at the time this disease was epidemic, and increased the confusion to which it gave rise.

Nothing at all really occurred that should be considered adverse to the efficacy of vaccination. On the contrary, no child who had been vaccinated, died; whilst, of 160 individuals who had not been vaccinated, and who caught what was really small-pox, sixty became the victims of this disease.

Various prejudices continuing to exist against vaccination in some parts of Germany, Dr. FISCHER, of Hildburghausen, was induced to investigate their origin; and he has published a report of the results of his enquiry, as relates to Hildburghausen and Eisfeld. (71) Those prejudices were found to be without a just foundation. Amongst those which he notices, was an opinion similar to one that has been advanced in England: that, since vaccination has been practised, scarlet-fever and measles have been more fatal than formerly. Dr. Fischer considers that this arises solely from so great a number of children living to be subject to them when prevalent, who but for cow-pox would have died in early infancy. Those diseases are not, he says, individually more severe.

Failure in the prophylactic power of vaccination has sometimes ensued, apparently in consequence of many practitioners having been accustomed to collect the virus as early as the sixth day. Dr. Fischer has often deferred doing this until the ninth or tenth day; and has, indeed, frequently employed the scab, moistened when required for use, with the most satisfactory results.

The report addressed to the marine department in France, by M. le BARON DONZELOT and le COMTE DE LARDENOY, states, that small-pox has not been witnessed at Martinique during the last ten years. The number of persons vaccinated there amounts

---

(71) *Kurze Geschhichte der Vaccination im Herzogthume Hildburghausen und der Impfung, im Jahre* 1818, *in den Amtern Hildburghausen und Eisfeld; mit einigen Allgemeinen Bemerkungen über di se Krankheit.* Von dem Geheimenhofrathe und Leibartze Dr. FISCHER. Altenburg und Leipzig *Allgemeine Medizinisch Annalen.* Erstes heft, 1819.

to fifty thousand. Guadaloupe has been equally free from that disease.

Professor PUERARI, of Copenhagen, observes, in a letter, that small-pox has been totally unknown in Denmark since the year 1810. This entire freedom from it appears to depend on the isolated situation of that country, and the almost universal vaccination of children with the cow-pox, in early infancy.

*Hydrencephalus* affecting children is a disease of such frequent occurrence, and so destructive in its nature, that no writer treats on the subject without preliminary lamentations on those circumstances, and on the ignorance we are in respecting the more general and efficient causes of its production. Numerous treatises on this malady have within the last half century been produced, all of which have left much knowledge of the first importance regarding it to be acquired, although but few have not contributed somewhat towards the filling-up of the dark chasm: and, pursuing our course, "*floriferis ut apes in saltibus omnia*," we have collected what we believe will, in no small degree, contribute to the same end. The first extracts we adduce with this view, are from a work by Dr. BRACHET, of Lyons. (72)

Dr. Brachet proposes the term *hydrocephalitis* as a designative epithet for this disease, as showing by its etymology the nature of the affection; and, since ideas of things will, in persons in general, be influenced by the distinct signification of the terms applied to them, it may be useful to adopt the term.

We pass over the author's exposition of the history of the malady and its causes, (since it is hardly more certain and precise than that of former writers,) until we arrive at his remarks on the effects of the customary tight and confined dress of children. This, Dr. Brachet observes, by preventing the free afflux of blood to the surface, throws it on the internal organs, especially the brain, which is the only one immediately free from the pressure it produces. The thorax, being compressed, executes but imperfectly the movements proper for inspiration: the whole of the dark blood cannot traverse the lungs; the right cavities of the heart thence become distended; a reflux of blood takes place in the veins; this fluid stagnates in the different organs, and produces in them alterations which are the necessary results of the presence of too large a quantity of this fluid, and especially of blood become improper for the support of vital action. The delicate texture of the brain, and its vital activity, expose it more than any other parts to the deleterious consequence of this influence. If the effects are prompt, the

(72) *Essai sur l'Hydrocephalite, ou Hydropisie aigüe des Ventricules du Cerveau.* Par J. L. BRACHET, M.D. de la Faculté de Paris. 8vo. pp. 208. Paris, chez Gabon.

infant perishes of apoplexy. The too numerous points of contact on the sensible skin of the new-born infant, have likewise often brought on a state of erethism, that has terminated in convulsions. In the infant, all is mobility; the tight dress opposes these rapid and incessantly-varying movements; it makes vain attempts to surmount the obstacle. These ineffectual efforts, and the vexation they produce, increase at the same time the irritation of the brain, and the afflux of blood to that organ.

Of all the divisions of hydrocephalitis which have been adopted, that of M. BAUMES is the best, in the opinion of Dr. Brachet: thus, he admits an acute and a sub-acute form. The course traced by WHYTT serves as his guide, and he adopts, with that author, the three degrees or periods which observation led him to describe.

Amongst the symptoms of the disease, he notices one, which we think almost decisively pathognomonic. If any liquid be offered to the child, it seizes the vessel with avidity, and appears, by a reiterated sucking motion of its lips, to show that it has constant desire for drink; or else its wandering imagination makes it perceive objects which it fancies it can seize; for, if the motions cease, and the lips be slightly touched, they are immediately elongated, and the sucking or drawing-in movements re-commence. The author establishes three varieties of the disease.

1°. *Nervous hydrocephalitis.* This occurs principally in children of excessive nervous mobility, or who have suffered attacks of epilepsy or other convulsions. Moral affections, the author thinks, are very frequent causes. In this variety, besides the symptoms which characterize the disease in general, the nervous phenomena are carried to such a degree of intensity as often to cause the others to be overlooked. The malady (long announced by startings during sleep, terrific dreams, and an extraordinary susceptibility to external impressions,) sets in with convulsive movements, which nothing will calm, and which continue almost unceasingly. The anguish is inexpressible, and the pains in the head intense, particularly during the irregular periodical exacerbations; there is constant agitation; the eyes fly from the light and roll in their orbits; the whole of the body, though sometimes only a particular part, is in perpetual convulsive motion; and sometimes there is hemiplegia from the onset of the disease. The urine is clear, and rather copious; vomiting is very distressing, because of the great efforts with which it is accompanied, though hardly any thing is ejected from the stomach; but its recurrence is, in general, not very frequent. The coma is not very great, and never complete; delirium is often absent; the convulsions succeed each other rapidly for a long time, and terminate by inducing, during a paroxysm, the catastrophe of this lamentable scene. This variety is much more irregular in its course than the other forms; it hardly stops to mark the three periods, and may terminate in a few hours, or continue for several weeks.

2°. *Inflammatory hydrocephalitis.* The author endeavours to justify this strange epithet, by saying that the terms inflammatory pleuritis, &c. are frequently used; but such incorrect expressions should not be followed. He means by it to signify that the inflammation is more intense in degree, and more isolated from any accessory circumstances. Its symptoms are, redness of the face, fixed pain in the head, extreme sensibility of the eyes to light: fever and heat are the precursory symptoms, but they never long precede the invasion of the former, and the disease often shows itself very suddenly. The exacerbations are here particularly violent, and the fever intense; the carotid and temporal arteries beat so forcibly as to be evident to the sight; the face is sometimes flushed on both sides, or alternately on one and the other; there is almost continual moaning: indeed, the whole combination of symptoms announces a state of active inflammation of the brain. Vomitings next appear, independantly of any sordes in the stomach. Stupor succeeds to the excitation, and it is not long before coma ensues. There is very frequently delirium, proportionate to the intensity of the malady. Convulsions and paralysis are more tardy in their occurrence, and less severe, than in the preceding variety. This disease follows a regular course, and presents to the accurate observer the exact succession of three stages: that is, if the inflammatory state, carried to the highest degree, does not abolish suddenly the vital actions, then, a few hours are sufficient to lead to death.

3°. *Gastric hydrocephalitis.* Frequent occurrence of indigestion; a state of idleness of the stomach; uneasiness about the præcordia; slight efforts to vomit, or even frequently repeated vomiting, especially after food has been taken; are, with startings during sleep and other convulsive movements, the preludes to this variety. This state may continue for a long time before the signs proper to hydrocephalitis manifest themselves: at length, the accession of vomiting is more frequent; the tongue becomes white and foul; pain in the head appears, acquires more intensity, and alternates with fits of vomiting, which sometimes suffer no interruption, and recur on the slightest motion, especially when the head is raised and unsupported. During the second period, they are somewhat calmed. All the vital powers seem to be concentrated in the affected organ; the general excitation ceases, and a deceitful calm ensues, against which the practitioner should be on his guard. However, the irritation of the stomach is only concealed, and it soon after re-appears with all its former severity. We have seen patients not experience a moment of repose, the stomach being constantly agitated by continued convulsive efforts to vomit. It is in the midst of a paroxysm of this kind, that the patient's sufferings terminate with his life. The other signs of hydrocephalitis keep an equal pace with the vomiting; but this is so frequent, so distressing, and announces a gastric affection so intense, that it almost solely engages the attention of the physician.

Dr. Brachet thinks the above distinctions of the highest importance, although each of these varieties is not often present in so clear and exclusive a manner.

The observations relative to the semiology of these affections are good, but we cannot indulge much farther in our extracts from this work: those shall, therefore, be such as relate to the evidences furnished by dissection; which we give in this place rather than in the section on Pathological Anatomy, for the convenience of contemplating them at the same view with their symptoms and signs. Dr. Brachet found the arachnoid membrane opaque and thickened in several points, sometimes presenting the redness of inflammation; in one case, only a little pus was diffused over its surface. The pia mater was thickened and tumid, and infiltrated with almost concrete albuminous matter. The winding grooves between the convolutions of the hemispheres of the cerebrum, were less deep than ordinary; the brain, more contracted in bulk, appeared also firmer, and its medullary substance was to the touch less viscous and filamentous. The arachnoid distributed in the ventricles was very evident in many places; and in one instance was covered with an almost membraniform, albuminous, concretion. The ventricles, much dilated, ordinarily contained about two ounces of limpid, colourless, serum. In general, the cerebral vessels were much developed, and in a state of turgescence. In three cases, Dr. Brachet examined the abdominal cavity, and one only presented well-characterized traces of intestinal inflammation: in this instance, enteritis had preceded the invasion of hydrocephalitis. The observations forming the basis of this abstract, with remarks on the accounts given of the evidences furnished by dissection, by Whytt, Fothergill, Watson, Cheyne, Rosen, Odier, Bard, Ducasse, Itard, and Coindet, constitute this section of the work.

Dr. Brachet's theory of the disease is lucid and satisfactory, and founded on the best principles of modern physiological pathology. The reader is requested to admit this expression.

In thus passing over the treatise of Dr. Brachet, it has been our object to seize, though often in an isolated manner, those observations which may furnish useful ideas for reflection; and serve, in a certain degree, as indications for the direction of the course of other investigators: we have not made the vain attempt to give, in a brief abstract, a full view of a concise and methodic dissertation.

The observations of Dr. YEATS on the same subject (73) are now to engage our attention; but, being adduced only as additions to his former remarks, they will not detain us long. The intention of Dr. Yeats is to show that disorder of the gastric system is a frequent cause of water in the brain; a circumstance that, perhaps, is not sufficiently attended to by practitioners in general, although so forcibly pointed out by Dr. CHEYNE: but, the remarks of Dr. Yeats would inculcate this in too general a manner, probably much more so than he is himself advised of, or really wishes to effect. In pointing out what he considers to be an important view of the subject, and not at the same time sufficiently noticing its other relations, his propositions convey too partial and exclusive ideas of it to the mind of the reader. That hydrencephalus sometimes, and probably in the greater proportion of cases, is a consequence of previous disorder of the stomach and bowels, is sufficiently evident; but it is also equally certain that it has often occurred as a primary affection of the brain. The remedial measures indicated by those different views of the disease are so dissimilar, and the consequences of each of them so deleterious to the patient if erroneous, that too much caution cannot be exerted in forming the distinction, if it really exist.

Dr. PORTER, of Bristol, (74) has made some interesting remarks on this subject. "An inflammatory condition," he says, "is not always extended to every set of vessels, or even to all the branches of the same vessel, with which a part is furnished." This is an established fact, the particular observance of which in this, as well as in many other important cases, is in a great degree dependant on the principles of what may be termed physiological pathology. By pursuing the course pointed out by the above principle in its application to hydrencephalus, Dr. Porter has been led to conclude, that in this disease "the fatal link of the chain of morbid action consists of an inflammatory condition of the posterior arteries of the encephalon. I say, the *fatal* link; the link on which the fate of the patient is suspended, and to which our attention should be directed; not the *mortal* link, by which the poor sufferer is dragged to the grave, and on which Darwin had his eye when he wrote *Trephine?*" In support of this hypothesis, Dr. Porter observes, "In hy-

(73) *An Appendix to the Pamphlet on the Early Symptoms of Water in the Brain*, &c. By G. D. YEATS, M.D. Fellow of the Royal College of Physicians, in London, &c. 8vo. pp. 97. Burgess and Hill, London. 1819.

(74) *Medico-Chirurgical Journal and Review*, No. iii.

drencephalus, as such, the flushed face; the highly irritable iris; the frantic delirium, manifesting an inflammatory state of the fascial branches of the external carotid and its meningeal arteries; are not symptoms necessarily present: they are only so when phrenitis is superadded: for it is an inflammatory state of the meningeal artery that produces the symptoms which range themselves under this term." "The extension of inflammation from one texture to another contiguous, obtains everywhere; but the confinement of inflammation to particular textures and to particular vessels, previous to such extension, and very frequently without such extension, is equally true."

As far as Dr. Porter's observations have extended, dissection has shewn the correctness of those remarks: signs of inflammation were always most strongly, and sometimes solely, evinced about the basis of the brain, especially in the tuber annulare. Yet it is certain that, in some cases of hydrencephalus, it is the membranes of the hemispheres of the cerebrum that are almost exclusively the seat of the inflammation which gives rise to the lymphatic effusion. Dr. Rush, of Philadelphia, indeed, attributed the ordinary species of it expressly to inflammation of the arachnoid membrane; and, with respect to phrenitis, Dr. Portal, (75) when treating the question, whether or not a distinction can be made between inflammation existing in the substance of an organ, and that which affects only its enveloping membrane, (which he decides in the negative, as regards those organs which receive tunics from the peritoneum,) says, "Phrenitis, the seat of which had been fixed in the membranes of the encephalon, was said by Coiter, from observations illustrated by dissection, to arise from inflammation of the intimate structure of the brain, and not of its enveloping membranes: an opinion that has been confirmed by Morgagni, and by many other anatomists." The coincidence of the opinion of M. Portal with those he here adduces, and the assertion of Rush respecting hydrencephalus, with the observations of some other eminent writers, are powerful obstacles in the way of Dr. Porter's hypothesis. The symptoms and signs of phrenitis and the ordinary form of hydrencephalus, seem however to show the correctness of the views of this physician. It is a question which we hope to see assiduously investigated, for it bears an important relation to the appropriate treatment of those diseases; and will probably lead us to distinguish, more accurately than has hitherto been done, the distinction between external and internal hydrencephalus.

There are some varieties of disease respecting which accurate pathological knowledge is not of essential importance, since, if

---

(75) *Journal Universel des Sciences Medicales,* tome xiii.

the treatment ordinarily employed be not strictly appropriate, it at least does not produce absolute injury. Thus, stimulants applied to the stomach have been beneficial in inflammation situated in a remote part, by acting as counter-irritants; though, perhaps, a more judicious observer, who had ascertained the real nature of the affection, would have employed depletory agents. This, however, is not the case when the alimentary canal is the seat of disease. If stimulants are here improperly used, they can only prove deleterious. The tracing, therefore, what has generally been termed idiopathic fever, to inflammation of the mucous membranes of the gastric system, is probably the most important of the modern discoveries in pathology. The ordinary forms of dyspepsia, and dysentery especially, are now well ascertained to partake of the same inflammatory character. On the latter subject, we have a mass of decisive evidence, in a work by Mr. BAMPFIELD; (76) in the contemplation of which the general practitioner will find much matter for reflection, that will be applicable to a variety of forms of disease of daily occurrence. Those whose scene of action lies in hot climates will meditate on it with particular interest, and derive from it much very important knowledge.

No species of disease has received more brilliant illustration from the present mode of pursuing the study of medicine, than dropsical affections, by the researches of Drs. BLACKALL, ITARD, CHEYNE, (77) PERCIVAL, (78) STOKER, (79) ABERCROMIE, (80) and JOHN CRAMPTON: (81) but, as a view of the highly valuable observations of the latter was given in our last volume, we shall not at present recur to the subject in a particular manner. Some notice of it, however, was necessary in conformity with our plan; and we have chosen to give a series of references to the progressive views of the subject, for the convenience of the student.

Until the recent discoveries respecting the structure of the brain, and the opinions respecting the relation of distinct portions of it to distinct functions, had enlightened our views, that deranged manifestation of the intellects, described by Horace in his admirable picture of the citizen of Argos, (82) and to which

(76) A *Practical Treatise on Tropical Dysentery, more particularly as it occurs in the East-Indies; illustrated by Cases and Appearances on Dissection, &c.* By R. W. BAMPFIELD, Esq. Surgeon, &c. 8vo. pp. 352. London, 1819. Burgess and Hill.

(77) *Dublin Hospital Reports*, vol. i. p. 269.

(78) *Ibid.* p. 293.

(79) *Transactions of the Association of the Dublin College of Physicians*, vol. i.

(80) *Edinburgh Medical and Surgical Journal*, April, 1818.

(81) *Trans. Assoc. Dublin Coll. Phys.* vol. ii.

(82) ——Fuit haud ignobilis Argis,

Qui se credebat miros audire tragœdos; persons of the finest genius are so especially disposed, (83) was, of all the varieties of insanity, the one most involved in obscurity.

Some curious cases of this hallucination, which contribute to show the truth of the opinions to which we have alluded, have lately been witnessed by Dr. Esquirol. He publishes them now, (84) he observes, as texts for some reflections on a future occasion.

The first of those cases discloses all the principal circumstances that render this affection so peculiarly interesting to the contemplative physiologist; and, as the narration of it is concise, we will give it in detail. It is, indeed, a portrait of all cases; just as the sentiments of Shakspeare were said by Jonson to be of all ages and of all time.

"M. ***, aged 43 years, of a sanguine temperament, prefect of a large city, on being falsely accused of treason, considered himself dishonoured, since he was suspected to have violated his duty, and cut his throat with a razor. He was carried to a neighbouring town. Cured of his wound, he persuaded himself that he was surrounded by spies. He is the more fortified in this belief, by his hearing voices which tell him to mistrust his servants, for they have betrayed him. These voices accuse him of being a traitor, and repeat, that, being dishonoured, he cannot do better than destroy himself. The voice of a lady, which he can well distinguish, tells him to have courage. These voices use by turns all the languages of Europe which are familiar to him; he hears them as distinctly as if persons were present: he has some difficulty to understand them when they use the Russian language, which he himself speaks with difficulty. He often goes into some retired place, with a view to hear them more freely; he often replies to them, often questions them, defies them, provokes them; they, on the other hand, excite him to anger. These voices make themselves heard a few minutes after he awakes; sometimes they waken him, or prevent him going to sleep. He is convinced that his enemies, with the aid of conductors, of mechanic means, can arrive at his most secret thoughts, and transmit to him the reproaches, the menaces, and the sinister counsels, which they confer on him. In other respects, he reasons admirably, and

---

In vacuo lætus sessor plausorque theatro:
Caetera qui vitæ servaret munia recto
More: bonus sanè vicinus, amabilis hospes,
Comis in uxorem: posset qui ignoscere servis,
Et signo laeso non insanire lagenæ:
Posset qui rupem et puteum vitare patentem.

(83) For instances—Tasso, Pascal, St. Theresa, Swedenbourg, Harrington, and the author of Æmilius.

(84) *Journal Génerale de Médicine*, Mars, 1819.

behaves with the most perfect politeness. He passes the summer in a castle, and receives many visitors, in order to distract his attention. When conversation interests him, he ceases to hear the voices: if it languishes, he hears them, he quits the society, and goes into retirement, in order the better to hear these perfidious voices, which begin to intrigue with him. The ensuing autumn he came to Paris: the voices still follow him; they repeat, that he should kill himself, and not outlive his dishonour; but he wishes to wait, that he may be justified. He visits the minister of the police, who receives him with kindness, and gives him a flattering letter, adapted to restore his peace of mind. He leaves the minister satisfied; but the voices again almost immediately beset him. I went to his hotel. He was walking in the court, and often around the edge of a pit. He received me with politeness, and assured me that he was not ill. On the following day, I had the same reception. In the course of this day, he is more agitated. He goes to the residence of a friend to confide to him his daughter, from twelve to fourteen years of age. In the evening, after having returned home, he continues to be agitated. The following morning, at daybreak, he goes to the prefecture of police, to make a declaration that he has a daughter, to whom he desires to bequeath the memory of her father free from blemish; and that he will not give way to those who urge him to destroy himself, until he shall be fully justified. The same day he is confided to my care.

"During a month, he remains alone in his apartment, eating but little, sleeping only a few hours, and walking about hastily like a man deeply occupied. He refuses all sorts of remedies, because he is not ill. His appearance is good, with respect to flesh, his countenance florid; he behaves with politeness, and reasons very well. After a month, he seems to desire that I should prolong my visits. I venture to speak to him of his malady, and of the motives for his confinement. He gives a full account of the causes of his present situation, and of the voices which incessantly pursue him. He listens to my arguments, but is not persuaded by them. The political changes, the approach of the European powers towards Paris, appear to him fables, invented in order to sound his opinions. Towards the end of March, 1814, after a long conversation, I invite him to visit me, that he may assure himself, by inspection of my books, that I should be a physician: he gives me a refusal. But three days afterwards, believing that he should take me unawares, he proposes to come to see me in my study: I immediately accede to it. After having looked over my books for a long time, he said to me, 'If these books are not purposely placed here, this library is that of a physician.' The siege of

Paris occurred a few days afterwards: he remains convinced that it was only an exercise of the cannon at a review of the troops. On the king being proclaimed, I sent to him the newspapers stamped with the arms of France: he reads them with interest; but he believes them to be suppositious, and that I had got them executed in order to sound his opinions. This he frankly told me eight days afterwards. I then offered to convince him, if he would walk out with me into the streets of Paris: this he refused. But, on the 15th of April, he suddenly said to me, 'Let us go out?' and we instantly went to the Jardin du Roi, where he saw the troops of the different nations. He then pressed my hand, saying, 'I see very well that you have not deceived me. I was ill, I am now cured: I need see no more; I am convinced.' We returned home; and from this moment the voices spoke to him less frequently: the following morning, he did not hear them at all. He was persuaded that it had been a nervous affection. He had some leeches applied, and put himself on a course of mineral waters. After a month, he went into the country, where he enjoyed perfect health, notwithstanding some severe distresses *(chagrins violens)* he experienced. The following year, he lost his only daughter, who constituted his hope and his happiness. He has returned to his native country."

The patient of the second case was a woman. Here the affection was also induced by moral causes; but was accompanied with more evident cerebral disease, and at length, and before the most confirmed stages of the hallucination, with continued fever of very long duration, attended with delirium. In this case, the hallucination consisted in visions of the objects of religious worship, conversations with the Deity in the human form; notions that she was chosen to prophesy, and, although she had led a dissolute life, to convert the morals of the people. M. Esquirol relates three other cases, the subjects of which were also females, in which the hallucination was of the same character. The immediate development of the affection was in these also apparently dependant on moral influence, but it had been preceded by organic disease. The promised reflections of M. Esquirol on this subject shall not be lost to our readers, and we expect in them much matter of deep interest.

Before this subject is resigned, we should give a reference to the memoir of Dr. Klapp, of Philadelphia, on "Temulent Diseases," (85) as it contains some observations well illustrative of the influence of morbid affections of the abdominal viscera on the functions of the brain.

Some interesting observations and reflections by Dr. Aber-

---

(85) *London Medical and Physical Journal*, vol. xli. p. 172.

CROMBIE, relative to the nature and origin of apoplexy and paralysis, were noticed by us on a former occasion: (86) we shall now complete the view we can give of them, by adducing some illustrations of the arguments then advanced.

The rapidity of the change from paralysis to apoplexy, and *vice versâ*, and the want of any evident morbid appearance on dissection, in many fatal cases, (excepting, perhaps, a little serous effusion,) support the opinion of Dr. Abercrombie, that those affections depend on some transient cause, and apparently on that which he has imagined,—an obstruction to the freedom of the circulation in the brain. He has also been led to suppose that many old cases of palsy, in which the patient has continued for years without improvement, though otherwise in tolerable health, and has at last died of some other disease, are connected with some disordered state of the circulation in the brain, or with a state of disease which is not organic. "Among the causes of these affections, have generally been reckoned," he observes, "cavities in the substance of the brain, containing the remains of former extravasations of blood; and some writers have contended, that extravasated blood may lodge in this manner for a long time in the brain, not only without being fatal, but even without producing any urgent symptom, after the removal of the original apoplectic attack." "I consider these important questions as still undecided." The arguments for the affirmatives to those questions are very forcible; and, in admitting the correctness of Dr. Abercrombie's views respecting the influence of disordered circulation alone, in many cases, we must not forget the disposition which all men have to apply original conceptions in too general a manner.

"An important circumstance in the history of paralytic affections is," says Dr. Abercrombie, "that many of them are connected with a state of the brain which is not apoplectic, but inflammatory." This proposition is illustrated by several observations; and, when speaking of the signs of this form of disease, he remarks, "Many of the cases are accompanied by convulsion, which sometimes appears on the same side with the paralysis, the convulsion attacking first and leaving the part paralytic; and sometimes they appear together, the convulsion on the one side, and the paralysis on the other. Many of them, again, are remarkable from not passing into coma, the patient remaining entire in his intellect to the last, or until a very short period before death; others, however, pass into coma at an early period. In some cases, the pulse is frequent; but, in others, it resembles the pulse of apoplexy." "This course of

(86) *Loco citato*, p. 31.

symptoms constitutes a case remarkably different from the attack of the apoplectic hemiplegia, which is generally sudden and complete." "A most important circumstance in the history of the inflammatory paralysis is, that all the symptoms may take place while the disease in the brain is in the state of simple inflammation, and that it may not have advanced beyond that state while they go through the usual course, and terminate in fatal apoplexy."

We have passed over many facts which are related by Dr. Abercrombie to support the foregoing arguments, since the limits of our essay oblige us to confine our extracts to but little more than his propositions; but, when the talents of this physician are considered, the insufficiency of such a view of the results of his observations will not appear so forcible as it would be, were we giving an account of the statements of a less accurate observer. Pursuing this method, then, we adduce the remarks of the author relative to the termination of the morbid affection that was the subject of our last citations. They are principally these:

"1°. It may be fatal, as we have seen, in the inflammatory stage, with all the symptoms of perfect apoplexy. 2°. It may be fatal by suppuration; and this may be either in the form of the encysted abscess, or of that extensive undefined suppuration which has been called sphacelismus cerebri. 3°. The inflammation may subside, leaving induration of a part of the brain, and thus producing permanent paralysis. This state of disease may continue a long time without being fatal."

"Many paralytic affections depend upon diseases of the spinal marrow."

"A paralytic state is known to follow severe rheumatism. It may be induced by long-continued cold, without rheumatism having taken place."

"There is a singular modification of paralysis which seems to be connected with the state of the circulation in the affected part." Dr. Abercrombie relates several curious cases apparently of this kind, and observes, that "they were probably connected with disease of the heart and great arteries."

The limbs thus affected were cold and without pulse, and the affected patients had visceral disease. We consider them to have depended on want of the due influence of the ganglionic system on the parts thus affected; the circulation consequently became suspended in them, (87) and from this a want of sen-

---

(87) We have on former occasions shown, that we adopt the opinion that the circulation in the smaller series of arteries is principally supported by the immediate influence of nerves on the vessels; and we also believe those nerves to be branches of the ganglionic system.

sibility in the cerebral system of nerves necessarily ensued. (88) Dr. Abercrombie's history of this affection powerfully supports our opinion: he says,

"This kind of disease may exist in a more limited degree, affecting only the vessels of a particular part of the body. In this case it is apt to terminate by extensive gangrene. *The limb is first affected with violent pain;* this continues for a day or two: *when it ceases, the arteries of the part affected are found to be without pulse;* and, after some time, it falls into gangrene."

But, Dr. Abercrombie is perhaps only more reserved in advancing *opinions* than we are, intending to confine himself chiefly to a relation of facts; for, that he has taken a view of this affection analogous to that which we have done, seems to be evident from the following remarks:

"There are singular facts upon record, which seem to indicate peculiarities of the circulation in particular parts of the body, probably originating in the relation betwixt the vascular and nervous systems. I know a gentleman, who, when heated by exercise, perspires over one half of his body and not on the other; the line being drawn with great precision from the forehead along the centre of the nose, and so downwards. When he is very much heated, the other side perspires also; but this only occurs from great exertions, or great warmth: the singular perspiration of one side, which I have mentioned, is a matter of almost daily observation. A child, mentioned by Dr. Falconer, became pale and emaciated on the whole left side of the body, without any evident diminution of muscular power; the right side remaining healthy. She recovered by warm pumping."

Observe, how forcibly this latter case supports the notion that the action of the small arteries, and consequently the nutrition of parts, depends expressly on a system of nerves distinct from that of the brain: those functions were here nearly abolished, whilst the voluntary power and its effects remained unimpaired. The paper of Dr. Abercrombie contains a multitude of observations, that tend more or less to confirm the foregoing opinion; and, although many of them are commonly known, we extract the following, in order to give a coherent view of this interesting subject.

"In some cases of palsy, there is loss of motion without loss of feeling." "Increased acuteness of feeling in paralytic limbs, has also been observed." "When paralytic limbs are recovering, the recovery sometimes begins at the extreme parts of the limb, as the fingers and toes, and extends gradually upwards; and sometimes it begins in the parts next the body, and

(88) See *London Medical and Physical Journal*, vol. xli. p. 22, for the opinion of Dr. Broussais on this point.

extends gradually to the extreme parts. Paralytic affections often begin by affecting a very small part of the body, as one arm, or the hand only, or sometimes one finger; the muscles of the tongue, of one side of the face, of the eyelids."

Dr. Abercrombie concludes his pathological observations and reflections with some remarks on lethargy; but on this subject he has not adduced any original facts of importance. His opinion is, that "the state of the brain in such cases differs from apoplexy, but it is nearly allied to it; for it sometimes occurs as the prelude to apoplexy, or it may be left as a consequence of it, after every other symptom has been removed."

Two cases of epilepsy accompanied with somnambulism, possessing a remarkable degree of interest, were recently related by Dr. MARTINET, in a memoir read to the *Athenée de Medecine* of Paris. (89) These cases, in general, tend much to support the notions of Dr. GALL respecting the relation of distinct portions of the brain to certain intellectual faculties; and the application of those principles to dreaming and somnambulism made by Dr. SPURZHEIM; but those to which we now refer, illustrated those opinions in a very striking manner. We shall detail them on another occasion.

The theory of scurvy proposed by Dr. BALME, or rather that which he has renewed from GIRTANNER, should not be passed over unnoticed; but, since we have already given a detailed account of it, (90) this will not at present arrest our progress longer than whilst we observe, that it is considered by Dr. Balme as a disease consequent on the want of due excitement of the system; the principal effects of which are first and essentially experienced in the digestive organs, arising from the long-continued usage of the same species of food, whether distinctly animal or vegetable; and a want of variety in the physical and moral agents on the animal economy.

"*Is the asthma of old people a nervous affection?*" (91) is the title M. ROSTAN has given to a tract, containing some very interesting pathological observations on some of the most important organs of the human body.

To speak with propriety, all diseases, excepting those arising immediately from chemical and mechanical agencies, might be termed originally nervous; but this is not the point to which our views are here directed: it is to determine whether or not the group of symptoms ordinarily termed asthma, are the immediate results of deranged nervous influence.

Holding an office to a large establishment peopled with old

---

(89) *Bibliotheque Medicale*, Février, 1819.

(90) See *Medical and Physical Journal*, vol. xli. p. 515.

(91) *Memoire sur cette Question: l'Asthme des Vieillards est il une Affection Nerveuse?* Par M. ROSTAN. 8vo. pp. 30. Gabon, Paris.

persons, M. Rostan has been enabled to trace the disease in question during the course of many years, and to determine by examination after death the state of the organization. After having adduced, as specimens, the descriptions given of asthma by Cullen and Pinel, he details his own observations.

The first case was that of an idiot, 61 years of age, who was subject to periodical paroxysms of great oppression of breathing, which happened during the winter only, and attacked her in the night. In the summer, she felt herself very well. M. Rostan observed this patient during several successive years. A large aneurism of the left ventricle of the heart, and ossification of the parts surrounding the bronchiæ, were the appearances observed after death.

A woman, 74 years of age, had been asthmatic for eighteen years: on opening the body, he found an active (92) aneurism of the left ventricle, with ossification of the aorta. The fourth case was one where the symptoms of asthma seemed to depend on aneurism of the right ventricle, itself caused by deformity of the thorax.

Ossifications of the aorta, and active aneurism of the whole heart, were apparently the causes of the same symptoms in the patients of the third and fifth cases.

In the sixth, they were owing to chronic inflammation of the pleura, accompanied with active aneurism of the right ventricle of the heart. Five other cases offered the same symptoms during life, and analogous appearances after death.

The *Hospice de la Salpetriere*, where those observations were made, contains a multitude of asthmatic patients; yet, M. Rostan says, he never saw a case purely nervous.

It has been objected to the views here taken of the nature of the disease, that these cases commenced as nervous affections, which at length produced organic disease; and we are disposed to think that there is some truth in these observations. M. Rostan combats them, and endeavours to explain the sudden and periodical accessions of the symptoms on other principles. Whether or not these may be generally applied, may yet be a question; but, in proving that asthma, or periodical dyspnœa menacing suffocation, are often results of organic lesion, M. Rostan has supplied an addition to medical knowledge of great importance in its application to the practice of the art.

Proceeding in our course from diseases of the vital organs to those affecting parts of secondary importance, we first turn our attention to a treatise on *cancer*, by Dr. Rouzet; (93) and,

(92) See *London Medical and Physical Journal*, vol. xl. p. 443.

(93) *Rechérches et Observations sur le Cancer;* par F. J. Leon Rouzet, M.D. Chef de Clinique Medicale de la Faculté de Médecine de Montpellier, ex-Chirurgien aux Armées Françaises, &c. &c. 8vo. pp. 358. Gabon, Paris.

however impossible it may be to give a sufficient idea of this work in an abstract, (for it contains fewer opinions than observations,) we shall adduce some extracts, which, if they do not contribute much to our knowledge of the nature of the disease, will at least show how researches with a view to acquire it should be conducted. This treatise, as the epigraph would indicate, (94) is deduced from observations guided by the principles of what may be termed *philosophical epiricism;* and, until we are possessed of more precise and extensive knowledge of facts on the subject, such a mode of proceeding is certainly the most judicious. The general structure of the work is founded both on analysis and synthesis; and, at the termination of each proposition, the author adduces several observations, which either confirm or refute it.

After having given a general description of cancer, or rather indicated the symptoms that announce it, he traces the course of the malady, its effects on the economy, and its terminations. He then treats on the pathological anatomy of cancer; on fungus hæmatodes; the sense that should be attached to the words schirrus, cancer, and carcinoma: the relations of the disease to age, sex, temperament, constitution, mode of life, professional occupation, and climate, are next examined. Some more expressly theoretical reflections are then advanced respecting whether cancer is in its origin a local affection, or only the local symptom of a constitutional affection: he attacks the erroneous hypotheses that have been produced respecting its nature; he endeavours to ascertain if it affect animals, if it be contagious, and if it is possible that it may be hereditary; and terminates by an exposition of the mode of treatment.

We cannot enter into the consideration of each of those views of the subject, but must confine our extracts to some of the most striking observations and propositions.

We need not adduce the author's general description of cancer, since it does not differ from that of former writers; but we cannot help remarking how forcibly this shows the difficulties attending the application of general descriptions of disease: for, when treating successively of its appearance in different organs, he has been forced to modify all the former traits in such a manner, that hardly any of its original character remains. In this, he follows the best authorities; for no precise description of the essential character of cancerous degeneration, with respect to all the organs it affects, has yet been generally determined on.

After having described the progress of the disease, when speaking of a female who had one removed fourteen years since,

(94) Rerum eventa magis arbitror, quam causas quæri oportere, et hoc sum contentus quod etiamsi quomodò quidque fiat ignorem, quod fiat intelligo. CICERO, *de Divinatione*, l. ii.

he says, "Nothing yet indicates the approaching return of the cancer; but this delay does not assure M. le Professeur Delpech that it will not; for, solely from having recognized the cancerous characteristics in the tumor, he is convinced, that if this patient lives sufficiently long, she will necessarily experience a new cancer." This opinion is also that of all the best and most experienced surgeons.

An important question is, whether or not a cancerous tumor is capable of being dispersed? The reflections of M. Rouzet on this subject, are chiefly intended to show the difficulties attendant on its decision in the present state of our knowledge. He raises some well-founded doubts respecting the possibility of the dissolution, and on the metastasis of cancer. He thinks that it does not propagate itself; for example, from the breast to the cellular tissue, to the muscles, to the bones of the thorax, excepting when there is but little pain, and consequently, a slight influence exerted by the cancerous mass on the whole constitution. Patients, then, worn out by the insensible progress of a malady always of long continuance, do not die until a very late period, and of the utmost degree of marasmus. These observations involve opinions relative to what has been termed the cancerous diathesis, which will be noticed in their proper place.

The termination of cancer by gangrene, is next adverted to as a very interesting occurrence; and the author refers for examples of it, to the works of Le Dran, Vandenblock, Imbert, Fages, Arnaud, Richerand, Saint-Armand, Everard Home, and Vautier. He does not add any thing new, after the most recent researches in pathological anatomy, respecting the various accidental tissues that are found in cancerous tumors. The word scirrhus, he says, ought only to serve to designate the presence of one of those accidental tissues; and that of carcimona, should be rejected as superfluous. After this a question is proposed, whether cancer can be primitively developed in all the tissues of the economy? Dr. Rouzet, with all the best surgeons, replies in the affirmative.

The chapter on cancer as it relates to the different periods of life, the sexes, various constitutions, &c. contains much interesting matter in numerous observations; but extensive researches are yet requisite to determine much of importance on these interesting subjects.

We now arrive at the point to which we a short time since alluded. M. Rouzet thinks that cancer is "constantly, from its origin, the symptoms of a particular diathesis, of which neither the elementary cause nor the primitive seat are known. The existence of a *cancerous virus* in circulation with the fluids, is not admitted; but, adopting the opinion of Bayle, he says, "Without attempting either to explain or to define this disposition, which is, and perhaps ever will be, essentially unknown, we designate it under the term of the *cancerous diathesis*, or the *disposition to cancer*."

M. Rouzet, in reply to the question, is cancer hereditary? answers in the affirmative: meaning only, what is by no means precisely determined, that which is commonly signified by hereditary dispositions.

The palliative measures, to which M. Rouzet reduces the medical treatment, consist in attentions to hygiene, the use of opium, hyosciamus, belladonna, cicuta, and tepid baths, to relieve pain; and local and general abstraction of blood, and acetate of lead as a topic, to combat inflammation when it manifests itself in the tumor or the ulcer.

After some reflections on the circumstances which, in individual cases, either favour or contra-indicate the operation for the destruction of the affected part, the author remarks, that he considers the removal of a cancerous organ as adapted to destroy the local effects of the cancerous diathesis, and consequently the sympathetic effects on the whole system; and to suspend the further development of the diathesis for a greater or less length of time. This is all that can be expected from the operation. He decides in favour of the scalpel exclusively as the instrument, whenever it can be employed; excepting, however, cases of very superficial cancers, which may be advantageously attacked by the arsenical caustic.

The boundaries which must confine our course (and which, however much we may feel disposed to it, we cannot overstep,) are close in sight; and we have before us one work, amongst some others, of which to give an adequate idea would require a greater extent of space than we can here devote to it, and previous reflection that the recent period of its publication has not permitted us to indulge in; but which, from the view we have been able to take of it, appears to be one of the most interesting amongst the numerous highly valuable productions in English medical literature, to which the last few months have given birth. This is an *enquiry respecting the nature of tuberculated accretions of serous membranes*, by Dr. Baron. (95) Hitherto, the more we have meditated on this work, the more interesting and valuable it has appeared, and the less we have felt able to say to what extent are the views it leads, (for they increase as we continue to contemplate,) or to advance any thing as conclusions that may thence be formed. The author is pursuing some researches respecting analogous affections of the

(95) *An Enquiry illustrating the Nature of Tuberculated Accretions of Serous Membranes; and the Origin of Tubercles and Tumors in different Textures of the Body.* By John Baron, M.D. Physician to the General Infirmary at Gloucester. 8vo. pp. 307; with engravings. London, Longman and Co.

mucous membranes: when these are also published, we shall give an exposition of the whole. These, then, with an expression of the gratification we have experienced in the perusal of a work on so interesting a subject, characterized by accurate observance and severe and close induction, are the only remarks we shall make respecting it on the present occasion.

Our chief object in this part of our sketch, is to trace those original observations and combinations of ideas, which open either more extensive or novel views over the regions of medical science: we have neglected to do this with respect to those contained in the preceding work, because we have not yet, by due consideration, been enabled to discern them so well and so comprehensively, as to effect what we desire with sufficient precision. Several treatises, valuable in themselves, but more confined in their influence, must now be passed over unnoticed. Many of these will, however, be spoken of on a future occasion, when we can adduce from them observations that will illustrate any subject under immediate consideration: this will be bringing them into notice in the most favourable manner; and we shall thus avoid tiring the patience of the reader, and occupying the space we have to devote to this essay, by a long train of general references. Several works by German authors are however before us, which should be exempted from these remarks; especially that of Dr. Schaffer, from which we made an extract in a former part of this sketch: but, as we purpose to give in the subsequent volume of this Journal, an exposition of the recent progress and present state of medicine in that nation, we may now be permitted to pass over works, of which the limits of this sketch would not permit us to give an adequate idea.

An essay on some diseases of the lachrymal organs, by Mr. Mac Kenzie, (96) must not pass totally unnoticed. It comprises a concise account of a species of disease by no means understood with sufficient accuracy by practitioners in general: we shall, in the ensuing number of this Journal, give such a view of it, as will show the forcible claims it has on the attention of the surgeon.

§ IV. In the preceding section, we have connected a view of the evidences of disease in the dead body, with the history of the symptoms and signs of the morbid affection during life, in those instances where such a connexion was more particularly essential to a correct knowledge of the nature of either of those subjects individually considered, or to show the degree in which

(96) *An Essay on the Diseases of the Excreting Parts of the Lachrymal Organs.* By William Mac Kenzie, Member of the Royal College of Surgeons, and Lecturer on the Anatomy and Surgery of the Eye. 8vo. pp. 95. London, Anderson and Chase.

they mutually illustrated each other. We have now to direct our attention to those changes of organic structure which are particularly interesting in an isolated point of view. Before we enter on this subject, it will be proper that an essay by Mr. LOBSTEIN should be referred to, which, with a brief view of the progress of this division of science since the commencement of the 16th century, points out in a very lucid manner the sort of information it will afford respecting the nature of disease in general; and comprises also some judicious remarks on the spirit that should guide the enquirer in his researches. (97)

We shall, in the first instance, take a view of the evidences respecting the nature of fever, furnished by dissection of the dead body. The Report of the Hardwicke Fever-Hospital, by Dr. CHEYNE, (98) comprises some observations on this subject, of the highest interest. The number of *post mortem* examinations was about fifty; and, in every instance, the most decisive evidence of the existence of inflammation of one or more parts was observed. Either the brain or intestinal canal, except in two cases, had been the suffering organs; and in by far the greater proportion of cases, both. The evidences of inflammation of the gastric system without a corresponding affection of the brain, were less frequent than that of the latter unaccompanied by the former. Indeed, Dr. Cheyne observes, "I do not recollect a single dissection in which the remains of an excited state of the vessels of the brain did not appear; in which the surface of the brain was not in an inflamed, or rather sub-inflamed, state; as was demonstrable either from the state of the minute arteries, or from consequent effusions. *Inflamed* would, perhaps, be too strong a term to apply to a degree of vascular action, which in no instance led to the formation of purulent matter, and which, in only one instance of all the dissections which I witnessed, or were reported to me, ended in the formation of coagulable lymph."

This extract does not oppose our previous remark, which was applied to the more decisive signs of inflammation.

In some cases, no mention is made in the tabular reports of the dissections, (which were chiefly instituted by Mr. MACDOWELL, "on whose knowledge of anatomy and accuracy of description the reader may implicitly rely,") whether or not there were signs of disease in the gastric organs; they are therefore to be considered as having evinced none. As a corollary with this, we adduce an observation by Dr. Cheyne, that in some cases, tenderness of the epigastrium had existed a short time (a

(97) See *Journ. comp. du Dict. des Sciences Medic.* tome ii.
(98) *Dublin Hospital Reports*, vol. ii.

day or two or more,) previously to death; when, on dissection, "all the viscera of the abdomen had a healthy look." Dr. Cheyne seems to consider that an inflammatory condition of the parts had existed, which subsided before the cessation of life. He also particularly states, "Let me add, however, that in the dissections which I have superintended, of those cases of fever in which this symptom predominated, the inner surface of the stomach was always more or less inflamed." The same remark, as to the want of the signs of inflammation when the symptoms of such a state had been evident, is also made respecting the state of the lungs.

This evidence of almost constant co-existence of inflammation of the gastric system and the brain in typhous fever, accords with the observations made by Dr. Broussais. (99). We mention this because but few persons have had more extensive opportunities for observance of the patients of fever than this physician; and he has for nearly twenty years devoted a particular degree of attention to the subject.

In the two cases already mentioned, in which no evidence of disease of the brain or gastric system is noticed, that of inflammation of the mucous membrane of the lungs was very distinct. This often accompanied the same appearances of the former organs.

These observations were made on the bodies of the victims of the fever epidemic in Dublin, during the year ending March 1818.

We next relate one of the most curious and interesting facts furnished by pathological anatomy,—the change of nearly the whole structure of the heart into a mass of fat, interspersed with merely cellular membrane. This occurred to the observation of Dr. Cheyne. We shall detail the previous history of the case on a future occasion: at present it may suffice to state, that the patient was a man, 60 years of age, who used a luxurious diet; had been subject to gout, which had not lately occurred in a decided form; had suffered an attack of apoplexy two months before his death, from which he perfectly recovered. A short time previously to this, he began to feel pain in the left side, and palpitation or fluttering of the heart, with

(99) Dr. B. believes the latter to be generally consecutive on the former. We have already shown, that, when inflammation of the mucous tissue of the alimentary canal exists alone, it gives rise, according to the doctrine of this physician, to what is ordinarily termed idiopathic fever. (See the last volume of this Journal, in the *Exposition of the Doctrine of M. Broussais,* passim.) The coincidence which we have noticed was observed by Dr. Beddoes, and struck him forcibly as a fact possessing the utmost degree of interest. Some memoirs which he wrote on this subject, a short time previously to his death, were published in the twenty-second and twenty-third volumes of this Journal. See also, the various memoirs by Dr. Kinglake, which during the last few years have been published in this Journal.

cough and slight anasarca; both of which continued until the occurrence of the second fit of apoplexy, in which he died.

"There were not more than two ounces of fluid in the pericardium. The heart was about three times its natural size. The lower part of the right ventricle was converted into soft fatty substance; the upper part was remarkably thin, and it gradually degenerated into this soft fatty substance. The cavity of the left ventricle was greatly enlarged: the whole substance of the left ventricle, with the exception of the internal reticulated structure and carneæ columnæ, was converted into fat; the valves were sound; the aorta was studded with steatomatous and earthy concretions.

"The scalp was bloodless. The arachnoid membrane was slightly opaque; there was some fluid between it and the pia mater, and the vascularity of the latter was increased, more particularly over the middle and posterior lobes of the cerebrum on the left side, where, in a large patch, it was thickened, and of a deep-red colour. The brain was firm; its cortical substance of a pale-drab colour. There were between three and four ounces of fluid in the ventricles."

A case of obliteration of the abdominal aorta was witnessed a short time since, by Dr. Goodison, of Wicklow, in a female subject brought to the *Hospice de la Pitie*, at Paris, for dissection. (100) Unfortunately, no account could be obtained of the state of the vital functions of the subject during life. It was obliterated from the origin of the inferior mesenteric artery downwards for the remainder of its length, together with greater part of the iliacs on each side; the cavity of that on the left side being obliterated to its bifurcation into external and internal, and that of the right, to more than one half of the length of the common iliac. The artery lay close, and was firmly attached to the spine. It had precisely the appearance of the trachea; being rendered flat upon its posterior surface, but preserving anteriorly its circular, or convex, form.

The iliac veins were so intimately connected with the arteries as to cause considerable difficulty in their removal. There was a large quantity of gelatino-cartilaginous matter surrounding that part of the aorta and vena cava, together with the portions of the iliac arteries and veins which were included in the disease. The heart appeared of the natural size, with some firm and strong flakes of coagulated lymph here and there interspersed on its surface. The corpora sesamoidea of the semilunar valves of the aorta were considerably enlarged. In the mitral and tricuspid valves, the appearances termed "vegetations" by M. Corvisart were very manifest. The aorta, at its

(100) *Dublin Hospital Reports*, vol. ii.

arch, was expanded to nearly double the natural size; and studded, on the inner surface, with ossific matter. The obliterated part of the artery was almost completely ossified throughout. The arteries branching from the aorta, from the intercostals downwards, were considerably enlarged in their diameter. The arteries of both the superior and inferior extremities, appeared of rather diminutive proportion. The general appearance of the body could not be said to indicate any thing of disease.

The immediate cause of death appeared to have been inflammation of the right lung, in which three large abscesses had formed; one of which opened into the trachea.

At present, we only give a reference to some observations made by Dr. Colles, of Dublin, on the state of the parts connected with the head of the femur, consequent on fracture of the neck of that bone; which he relates in the Hospital Reports, already alluded to. They will be particularly considered on a future occasion.

Some information respecting the formation of hydatids may, perhaps, be derived from the following account of the morbid appearances observed in the body of a man, 22 years of age, who died in the New-York Hospital, of what was considered to be quotidian intermittent fever. (101). We wish the history of them had been more particular and explicit.

"On raising the sternum, the first thing which presented was the pericardium, so much enlarged and distended as to fill the anterior part of the chest, and completely to obscure the lungs on each side: the pericardium being opened, about a pint of fluid was discharged. The heart was found much larger than usual, and its anterior surface was covered with coagulable lymph, showing marks of inflammation; the coronary veins were also much enlarged, and distended with blood. An incision was made into the left ventricle of the heart, and there was found a shining yellowish body, nearly filling the cavity, and attached to different parts of it by little shreds of coagulable lymph, resembling sewing-silk. On examination, it was found to be a membranous bag, containing a semi-transparent gelatinous substance, resembling the fat of boiled beef. This having been removed, the valvulæ mitrales were found partly ossified, and partly cartilaginous. The heart was removed, for the purpose of a more favourable examination; and, on separating it from the vena cava, a similar substance was found in the right auricle: also, in all the blood-vessels from the heart into the lungs, and two in the aorta, extending as far as the

(101) *The Medical and Surgical Register; consisting chiefly of Cases in the New-York Hospital.* By John Watts, jun. M.D. Valentine Mott, M.D. and Alexander Stevens, M.D. 8vo. pp. 163. New-York, 1818; and Souter, London.

arch. The other cavities of the heart were opened, and a body of the same kind was found in the right ventricle. The left auricle was empty; the lungs were free from disease. Two substances of the same nature, about the size of a pigeon's egg, were found under the liver. The kidneys were unusually large, but had no appearance of disease: the other viscera were natural." This case is related by Dr. P. C. Tappen.

§ V. Thus far we have treated of medicine as a science: we have now to consider it as an art, by showing the application of the foregoing principles to the cure or the alleviation of disease; the first step towards which is *Semiology*,—the doctrine of the evaluation of the symptoms and signs of morbid affections.

As this arrangement of the objects of medical science differs from that adopted in systematic treatises, we have thought it prudent to commence each section with some remarks on the point of view in which the subject of it is here regarded.

The term semiology, applied to diseases, may be properly made to signify the doctrine, not only of what are expressly considered their *signs*, but also their *symptoms* and *phenomena.*

Every change that takes place in the body, evident to the senses, is a phenomenon: in disease, there is some alteration in these appearances; and, by observing the constant connexion between any certain forms of them and a certain state of the organs on which they depend, we come to consider the former as symptoms of the latter. Abstract ideas formed by the mind, may be constituted signs; and symptoms, though in themselves objects of the senses, may, by an effort of the judgment, be converted into signs: thus, a brown furred tongue and tremulous motion of the lower-lip, are signs of a disordered state of the stomach and disposition to vomit; but they are not symptoms of such a state. Symptoms are more immediate and sensible effects; they follow their causes, as Galen expressed it, just as a shadow follows a body.

Semiology, then, in its most comprehensive sense, comprises the doctrine not only of the nature of the present state of a disease, but also, in some degree, that of the stages through which it has passed, and its probable consequences: it thus includes *diagnosis* and *prognosis*.

An *essay on the diagnosis of erysipelas, phlegmon, and erythema*, by Dr. Wetherhead, (102) will first engage our at-

---

(102) *An Essay on the Diagnosis between Erysipelas, Phlegmon, and Erythema; with an Appendix, touching the probable Nature of Puerperal Fever.* By George Hume Wetherhead, M.D. Graduate of the University of Edinburgh; Member of the Medical and Chirurgical Society of London; Lecturer on the Distortions and Diseases of the Bones, &c. 8vo. pp. 72. London, Anderson and Chase. 1819.

tention: the subject has forcible claims on it; and we must confess, that these were rendered still more powerful by the epigraph to the work: "Artis magnum partem esse duco, posse quæ rectè scripta sunt speculari:"—Profound and comprehensive reflection.

Passing over the more determinate diagnostic signs of phlegmon and erysipelas, which the author exposes with great precision and conciseness, we stop at the important question,—whether or not the latter, when existing in a distinct manner, is ever accompanied with suppuration? Professor CALLISEN, Dr. Wetherhead observes, who, with Dr. CULLEN, has given an excellent description of erysipelas phlegmonodes, justly says, "Raro vel *nunquam* veram suppurationem admittit erysipelas."

SENNERTUS, LORRY, TISSOT, BURSERIUS, SAGAR, and MACBRIDE, are also quoted by the author, as expressing the same opinion; but that suppuration frequently accompanies erysipelas, he says, no one can deny. Whether this arises from an extension of erysipelatous inflammation into the texture most usually the seat of phlegmon; (103) or whether the topical irritation of one inflammation acts as a local stimulus, and thus excites phlegmon; is a question not indisputably apparent. Dr. Wetherhead favours the latter opinion; he says,

"Erysipelas seems a disease *sui generis*, arising most frequently constitutionally; phlegmon and erysipelas are not convertible: the pus, when evacuated, puts a stop to the phlegmonous action; whereas, the peculiar action of erysipelas seems protracted by the bursting or opening of the abscess; the wound does not heal in the usual way; pus, in a short time, ceases to be secreted; the sore contracts rather than granulates, and serous matter is discharged from the wound. Were it merely an extension of the same action, the symptoms in each texture should coincide in action, in duration, and progress, without any dissimilarity." This, we think, admits of dispute; and the case mentioned by Bichât, referred to by Dr. Wetherhead, will admit of another explanation than that which he is disposed to give of it. "Bichât says, 'un cautère établi au bras gauche cessa de suppurer,' when attacked with erysipelas; and that the erysipelas was repelled by re-producing the suppuration by an ointment made of basilicum and powdered cantharides. "This is a fact," Dr. Wetherhead continues to remark, "which goes strongly to establish the distinct natures of phlegmon and erysipelas; and tends to show, that they are not convertible; that suppuration is not natural to erysipelas; and that the phlegmon present is not an extension of the erysipelatous action." These

(103) "Phlegmon chiefly attacks the cellular membrane; erysipelas, the dermoid texture." p. 13. "The extension of inflammation from one of those parts to the other, constitutes erysipelas phlegmonodes."

propositions are nearly probable, but by no means established. The author is more determined in his opinions respecting the diagnosis between erysipelas and erythema. The part of the work devoted to this subject, possesses great merit: it evinces remarkable accuracy of observance, and comprises a very extensive series of facts, arranged with much judicious discrimination. We shall adduce some of its most striking traits.

Erysipelas and erythema have been considered by several eminent pathologists as one disease, varying only in degree; but Dr. Wetherhead, with CULLEN, WILLAN, and some others, is induced to believe them to be distinct in their nature, and to arise from different causes: the former being connected with some particular state of the system; the latter, merely a local affection: and he adduces many powerful arguments in support of this opinion. Erysipelas is ordinarily attended with fever, perhaps always when present to much extent; erythema is not connected with systematic derangement. We should observe, that Dr. Wetherhead considers erysipelas, "under certain circumstances, perhaps always, infectious;" and he refers to several observations (104) he had made some years since, which he thinks prove the truth of this notion. In erythema, there is no tumefaction. Erysipelas is always accompanied with more or less swelling; and this the author considers to be the most diagnostic symptom of this disease. This swelling arises from effusion of serum into the cellular membrane beneath the dermoid texture: it is also accompanied with lymphatic effusion into the cellular membrane lying over the cutis vera. The former gives rise to diffused tumefaction; the latter, to vesications, or bullæ: the latter, or vesications, alone attend erythema. Dr. Willan seemed to consider bullæ as the specific distinction of erysipelas; but Dr. Wetherhead thinks the effusion of serum into the sub-cutaneous cellular membrane, to be the most constant and characteristic symptom. Bullæ, he also remarks, differ from vesications only by the manner in which the fluid is poured out: when this takes place rapidly, the cellular septa are ruptured, and the fluid of many cells is collected into one cavity; when it occurs slowly, each cellule distends gradually, and thus distinct vesicles are formed. Erythema does not pit on pressure, for reasons which the preceding remarks will render obvious. "Œdema is synchronous with, and consequent to, the efflorescence of erysipelas; but, in erythema, the œdema, when present, *precedes* the cutaneous inflammation."

The observations already adduced are, we consider, sufficient to disclose some interesting and important views respecting the nature of the diseases that form the subject of them. They

---

(104) Published in the *London Medical and Physical Journal*, June, 1814.

will, at the same time, give an idea of the merit of the work whence they were derived. The essay of Dr. Wetherhead contains, also, some reflections on the etiology, pathology, and treatment, of those diseases, which are not noticed here, as being foreign to the view with which we entered on its consideration: the same remark will also apply to the appendix on the nature of puerperal fever.

The signs which appertain to the crises of diseases, are those least understood by general practitioners. It is not necessary to adduce theoretical arguments in favour of the disposition of any individual acute disease to run through particular changes in certain periods: this is admitted as a truth by nearly every physician of experience and accurate observance. The manner in which the subject should be regarded, was explained in the *Exposition of the Doctrine of* M. BROUSSAIS. We shall adduce some extracts from the Report of the Hardwicke Fever-Hospital, by Dr. CHEYNE, to illustrate those phenomena, as far as regards fever. His remarks relative to semiology in a more general manner, must be deferred until the whole report is subjected to a more particular review.

In April and May, affection of the lungs was the predominant symptom of the early period of fever: a lax state of the bowels and sleep, and then perspiration preceded by rigor, were the most frequent signs of the resolution of the disease.

The most perfect crisis during the summer months consisted of three stages: first, a state of restlessness and anxiety, with flushing of the face, rapid pulse, frequent laborious breathing, and increased heat of the surface, with great distress at the pit of the stomach from heat, tenderness, or pain; which distress was not unfrequently relieved by vomiting. The patients were in a state of universal uneasiness, which would have been truly alarming, had not its tendency been known; and this state sometimes lasted for the greater part of a day. Secondly, a rigor or tremor, not unlike the cold fit of an ague: the patients shivered and complained of excessive cold, although the thermometer, in contact with their body, stood at 105° Fah. This rigor seldom lasted long: sometimes, only a few minutes; at others, half an hour, or an hour. In the third stage, warm perspiration flowed from the whole surface of the body; and this generally completed the salutary phenomena. In some patients, the fever seemed to end in mucous diarrhœa; in others, free expectoration took place, with relief. Rigor was sometimes critical, even when not followed by sweat. In some instances, perspiration, with or without rigor, continuing for a short time, took place; other patients perspired for two or three days, with little or no interruption: in either case, perfect crisis was generally the consequence. But the effort at crisis by perspiration was not always effectual, till it was repeated several times on successive days, or successive critical days. In the greater proportion of these cases, symptoms of inflammation of the brain and lungs were most prevalent in the early stages of the disease.

From the middle of August until the end of March, the state of the epigastrium demanded constant attention. In three cases of four, the epigastrium was tender on pressure; sometimes remarkably so. The signs of crisis were very nearly similar to those already described. In many cases attended with alarming symptoms, crisis took place about the fifth or seventh day: in such cases, however, a relapse occurred very frequently about the end of the second week; and a second and final crisis took place on the seventeenth or twenty-first day, counting from the first invasion.

Some remarks on the diagnosis between *ascites* and *encysted dropsy* in the abdominal cavity, by M. Rostan, (105) which appear to be well-founded, and of obvious facility of application, will terminate this section. M. Rostan says,

"In the examination of dropsical patients, on using percussion of the abdomen, so as to produce fluctuation of the liquid, we have remarked, that, in ascites, this percussion, when made at the most eminent part of the abdomen, gave rise, in most cases, to a sound similar to that in tympanitis: we thence concluded that the intestines, distended by gas, floated above the fluid, where their specific gravity obliged them to rise. Having often had occasion to make a similar examination on persons affected with encysted dropsies, the development of which was considerable, (for it is only then that doubts can exist respecting the nature of the disease,) we have observed, on the contrary, that the fluctuation was very evident at the most prominent part of the abdomen; whilst the sound of stricken air took place at the sides, the most inferior parts, the patient lying on the back. The reason of this may be more perfectly conceived, on reflecting that the ovary, enormously distended, thrusts the intestinal mass beneath itself and to each side under the ribs."

We here conclude this subject, having noticed the most interesting observations which have been expressly advanced as relative to it. To collect all the dispersed remarks, would not be conformable to the plan of the essay; and, indeed, the most important of these have been necessarily involved in what has been related in the section on pathology.

§ VI. The Greeks, whose fervid imaginations led them to form symbolic images of the deepest physical and moral truths,

(105) *Nouveau Journal de Médecine,* tome ii.

designated Hygeia as the daughter of Æsculapius, the divinity of the medical art. But, the claims of physicians to the glory of being the patrons of that preservative genius, have been disputed by those who think that temperance and activity of body are the only true and efficient guardians of health. The latter opinion is, with some exceptions, well-founded; and the present neglect of such customs as tend to promote athletic exercises, is a general subject for regret with philosophical observers of human nature. Amongst the ancients, gymnastic exercises formed part of the daily habits of the senator, the magistrate, the soldier, and the philosopher; and the palestræ were, during the most flourishing periods of their history, under the superintendance of the most eminent of their physicians. The works of Hippocrates and Celsus will show how much importance was attributed to them, both as measures of hygiene and as remedies for disease; and the records of their historians will evince their beneficial effects, in their relations to the political importance of nations, and in their influence on the physical and moral constitution of individuals.

Since the establishment of the northern nations in the more temperate climates of Europe, gymnasia have been totally unknown; but, the warlike habits and exercises of those people prevented, for many centuries, the ill consequences that have since ensued from their destruction. Several modern physicians, amongst whom may be particularly designated Francis Fuller, attempted to effect their re-establishment as national institutions; but without success: the honour of first furnishing to modern Europe the gratifying spectacle of upwards of an hundred young men met for the purpose of pursuing a methodic course of athletic exercises, is solely claimed by an individual. But, as it appears from a report on a memoir lately presented to the Societe Medicale of Paris, (106) that M. Clias is about to publish an account of the system he has adopted in his academy at Berne, we shall defer, until the appearance of that work, the particular consideration of this subject; which may, perhaps, be made to appear more interesting and important to the physician than is generally imagined.

When we took up the pen to write this section, it was with an intention to relate some personal observations on the effects of this method of exercise; but the closing limits of this sketch admonish us against it. This brief allusion we shall, however, permit to pass; since it may serve to direct others to a source, whence many important indications relative to medical economy may be derived.

Next in importance to the measures above alluded to, are

---

(106) *Journal général de Médecine*, Fev. 1819.

those which relate to the prevention of contagious diseases; but on this subject we have nothing novel to produce: and, as before remarked, we must leave to the reader the development of many indications involved in the section of this sketch which relates to pathology.

Medical topography, when well executed, furnishes many inferences conducive to hygiene. On this subject, we have to notice a work by Mr. DOUGLASS, (107) which possesses a considerable degree of merit. The observations of the author were made during the late campaigns of the British troops in Upper Canada; and, should that country become again the scene of war, this work will, in the view we have already alluded to, be of eminent utility to the army-surgeon. The title which Mr. Douglass has given to the results of his observations and medical experience, does not indicate those of the most value, and which possess the most extensive interest; and it led us to defer the consideration of them until the present occasion, when we cannot extract many original pathological remarks, which render them worthy of the perusal of the general practitioner, as furnishing numerous ideas that, by reflection, would become extensively applicable.

The perusal of this work will afford much pleasure independent of that derived from the medical doctrine it contains. The scenic descriptions of an interesting country, the ideas it conveys of the habits and duties of the soldier during a campaign, and the slight sketches it furnishes of military events; give to it some of the charms of the "Relation Chirurgicale de l'Armée d'Orient;" and of the various works which we previously possessed, deduced from a review of analogous scenes.

§ VII. "There exists a species of medical knowledge, that is, in a manner, natural to man, which has been displayed in every nation, and cultivated everywhere with equal care. The same cause that led men to prepare themselves food and clothing, to construct habitations, to rescue themselves from the various accidents which in the course of nature must befal them, impelled them also to seek for means by which they could solace pain and alleviate disease: necessity was this powerful influence, and daily experience the means by which the ability to effect those objects was acquired. Such is empirical medicine. Our fore-fathers taught it to their children; generations have successively transmitted it from one to another; and the present is preparing it for the ensuing ages. It has had its epochs of glory, its partizans, and can show the monuments of its great

---

(107) *Medical Topography of Upper Canada.* By JOHN DOUGLASS, Assistant-Surgeon, 8th Regiment. 8vo. pp. 126. Burgess and Hill, London. 1819.

men; it long reigned solely over whole nations, and there are still many that acknowledge its precepts alone; it has rendered great and important services to mankind: and it has never ceased, nor ever will cease, to have zealous defensors, even amidst those sects of physicians that may appear to be most adverse to its existence." (108)

This was the description of empiricism given by a physician, whose works show the most philosophic views of the nature and objects of medical science. Nothing, however, can in reality be more indeterminate than the application of that term: if the subject be accurately investigated, it will appear that there is much difficulty in making a distinction between the mode of applying medical knowledge above alluded to, and that pursued by the most enlightened physician of the present age. Physicians of the present day do not, it is true, attempt to cure intermittent fevers by the mighty *Abracadabra;* nor surgeons, to heal a broken bone by the method of Cato: (109) but, if we observe the mode in which cinchona is ordinarily used in ague, the practice will be found to be not less empirical than the magical agency of Serenus. Both methods rest on the same basis: the observance of a constant, or very frequent, connexion or succession of certain series of phenomena, and of particular results from particular agents: the variance of their character consists only in a more or less accurate determination of the individual identity and mutual relation of the phenomena; and, consequently, more or less precision in the application of the agents employed to effect the desired results. The present refinement in the analysis of disease and the mode of action of remedies, has, then, caused the *Theriacas* of the ancients to be transferred from the physician to the hands of modern Circes: but, in avoiding the errors of our forefathers respecting the proper qualities of a multitude of medicinal substances, we are in danger of being deluded by generalizing too far our ideas of their mode of agency on the animal economy. Many physicians, whose talents claim respect for their opinions, say, that the number of the different species of the materia medica they require in practice hardly extends beyond twenty. There is in this notion a relic of Brunonism; and though, on superficial consideration, it may appear to be specious, the impropriety of it will be discerned when we reflect on the recent discoveries in physiology, particularly those which have determined the existence of a different degree of sensibility in each elementary tissue, as well as organ, of the system in the state

(108) BORDEU, *Réchèrches sur l'Histoire de la Médecine.* Liege, (réellement à Paris,) 1768.

(109) Incipe cantare in alto S. F. motas darrata dardaries astotaries, dic una pariter dum coëant.

of health, each of which will also individually vary in different forms of disease; and the evidence of almost every substance possessing a distinct mode of agency, and act ng especially on those parts of the animal economy to which its excitant powers bear a certain degree of relation. It was on these principles that Bichât intended to found his doctrine of the qualities of the materia medica.

These reflections are advanced to show to what an extent our observations in therapeutics would be prolonged, were they to comprise only those which immediately relate to the subjects treated of in the section on pathology; but, as this sketch is not intended for the elementary instruction of students, we have thought that it were better to indulge as far as possible in physiological and pathological observations, and leave the inductions from them to be formed by the reader. It is, then, only the most important treatises and dispersed reflections on the subject, that will here engage our attention; and particularly that of Dr. Welsh, on *the efficacy of blood-letting in fever.* (110) The nature of the subject, the extensive clinical experience whence the observations of the author are deduced, and the mode in which he has profited by it, concur to give to this work a particular degree of interest.

After having given an accurate description of the disease which forms the subject of this work, and a concise history of the use of blood-letting in fever, Dr. Welsh takes a particular and comprehensive view of every thing of importance relative to the application of the remedy, of which he so warmly advocates the use; and there is a character of precision in all his remarks, that will contribute much to favour the impressions which the results of his observations should produce. Let us consider the points that most forcibly indicate the utility of the practice which those observations decidedly inculcate.

A sophistic remark has frequently been made, even by physicians of considerable talents, that, whatever has been the treatment employed in fever, the mortality from it has not materially varied; and this has recently been expressly applied to the practice of blood-letting. This, Dr. Welsh proves to be erroneous, although, with respect to the remedy just noticed, it was a short time since, in a great measure, true; but, then, this arose from a reason already indicated by Dr. Rush: "This half-way practice of moderate bleeding has kept up the mortality

---

(110) *A Practical Treatise on the Efficacy of Blood-letting in the Epidemic Fever of Edinburgh, illustrated by numerous Cases and Tables, extracted from the Journals of the Queensbury-House Fever-Hospital.* By Benjamin Welsh, M.D. Superintendent of that Institution, and Member of the Royal Medical Society of Edinburgh. 8vo. pp. 208; with an Appendix of Cases, &c. pp. 150. Bell and Bradfute, Edinburgh; and Longman and Co. London. 1819.

of pestilential fever in all ages and in all countries." (111) It was not by acting thus, that Galen made the wondering people exclaim, "*O homo! jugulasti febrem!*" nor that a reduction of the mortality in the hospital at Edinburgh has been effected from the proportion of one in ten, to one in thirty. This, however, has been the result of the use of free venesection.

We shall first adduce the general conclusions of Dr. Welsh on the use of this remedy, and then some detached extracts for the illustration of its effects and the mode of its application.

"Copious blood-letting lessens the mortality in fever.

"It cuts short epidemic fever.

"Even when it does not save, it protracts, life.

"It mitigates all the uneasy sensations.

"It relieves irregular muscular action or spasm, subsultus, and singultus.

"It removes coma or delirium.

"It removes ischuria, constipation, and cuticular constriction.

"It removes oppression and morbid congestion.

"It reduces the temperature and pulse.

"It calms the respiration.

"It diminishes thirst, and improves the appetite.

"It often checks nausea, retching, and vomiting.

"It assists the operation of purgatives, and sometimes restrains diarrhœa.

"It conciliates sleep to the patient.

"It may be employed to five times the quantity believed till very lately, not only with impunity, but great advantage.

"Youth is no valid objection to its use.

"Old age is no impediment to its application.

"It is often of the greatest benefit in the most hopeless cases.

"It is scarcely of less utility in long continued, than in recent, disease.

"It alters the type of the fever to one more favourable.

"Where it does not cut short, it lessens the average duration, both of fever and convalescence.

"The utility of blood-letting in fever depends almost solely on its being copiously drawn; and it is on the merits of this principle alone, and more particularly its extension to a period of the disease, and under circumstances hitherto esteemed the most unfavourable for its use, that we rest our claims of originality, or distinction from the other authors who have recommended the application of the lancet in continued fever."

In the practice of Dr. Welsh, the average quantity of blood drawn from each patient, in 364 cases, in primary fever, was about twenty-four ounces, with the addition of what was taken

(111) *Medical Inquiries.*

by about twelve leeches; in relapses, twenty ounces, and eleven leeches: but, it must be observed, that several patients lost above one hundred ounces, and many more than fifty. One patient, a female, aged 25, suffered, at seven bleedings, an abstraction of 136 ounces, and recovered. Many patients had also been twice or thrice bled before their admission into the hospital.

The best time for taking blood, Dr. Welsh says, is in the evening, during the febrile exacerbation; as the patients then bear it better, and the relief next day is always observed to be more effectual and permanent. This observation accords precisely with those made by several other physicians of very extensive experience in the treatment of fever; especially Drs. CHEYNE, PERCIVAL, and ARMSTRONG.

We shall now take a rapid view of the objections that have been made to this remedy, and the replies of Dr. Welsh to them. They are—slow recovery, dropsy, phthisis, biliary irregularities, and relapse. The records of the hospital show the incorrectness of the first: the average time which patients remained there was about nineteen days. Dropsy, Dr. Welsh acknowledges, in the form of hydrothorax or ascites, is certainly an occasional consequence of excessive blood-letting: that is to say, *of blood-letting disproportioned to the disease.* In the cases he himself saw, none of the patients had been bled. (112)

"Phthisis," says Dr. Welsh, "is a consequence of blood-letting so very improbable, that I shall not waste any time in its discussion: ingenuity itself must be puzzled to discover the bond of connexion." He has never seen biliary irregularities distinctly referable to this source. The following expression respecting the last objection, is very emphatic: "Indeed, I shall never once apprehend the least danger from a relapse, whilst I know of the lancet as a remedy."

Leeches, Dr. Welsh says, are a most invaluable remedy in fever; and a similar observation is made by Dr. CHEYNE: (113) this physician, however, did not carry blood-letting to an extent at all equal to that in which it was pursued by Dr. Welsh. He is still an advocate for the practice, but many circumstances concurred to render it not so generally useful in his practice in Dublin; amongst which should be considered, the impoverished and exhausted state of a great proportion of the subjects of fever, previous to the attack of the disease. He, indeed, says,

"I seldom saw a patient early enough for the trial of free blood-letting as a means of arresting the course of fever: the

(112) See, on this subject, RUSH's *Medical Inquiries*, p. 304.
(113) *Dublin Hospital Reports*, vol. ii.

only individual with whom I attempted this summary method of cure, was one of our ward-maids. I was not able to superintend the experiment, which did not succeed. The quantity of blood drawn did not exceed twenty ounces,—a middle course, which I should not recommend any person to follow."

We shall terminate this subject, for the present, with the following remarks from the report of Dr. Cheyne; they indicate a multitude of truths, if duly reflected on: "During the winter and spring, I ordered blood-letting, sometimes with a view of abating re-action; generally, however, to subdue organic determinations; which being accomplished, the crisis followed, almost immediately after, in more instances than could have been expected: the result according with an observation which I believe may be found in Fordyce, that, when any one organ is much more affected in fever than the rest of the system, the whole disease will often abate as soon as the particular organic affection is subdued."

The more prompt and decisive good effects obtained from venesection in many severe acute diseases, has perhaps led us to neglect too much the benefits of a more immediately local abstraction of blood on various other occasions. In a memoir on the efficacy of local blood-letting in many cases where pain is a predominant symptom, (114) by Dr. Vaidy, of Paris, several cases are related, which evince the peculiar utility of this practice in a very forcible manner. In a case of severe sciatic neuralgia, affecting a young man assiduously devoted to study, for which all the ordinary measures termed general had been used without benefit, the application of thirty leeches on the limb produced much immediate relief; a second application was followed by more considerable benefit; and a third entirely removed the malady. Several other analogous cases, as well as those of gout, acute rheumatism, lumbago, muscular spasm, chronic pleurisy, syphilitic pains, &c. were equally benefitted by these means. A case of what the patient called wind in the stomach, relieved by the same measure, leads Dr. Vaidy to make some reflections on the real nature of the greater proportion of the affections thus termed; which is, that of chronic inflammation of the stomach, or of some of the contiguous viscera: an affection often led to a fatal termination, by the vulgar use of what are called carminatives.

In an acute cerebral affection in children, sometimes occurring suddenly, sometimes after the development of some precursory signs, evincing all the symptoms of nervous irritation and inflammation of the brain, with sanguineous congestion; showing, on dissection after death, more or less of cerebral lesion, the

---

(114) *Journ. comp. du Dict. des Sciences Medic.* Avril, 1819.

veins and the sinuses being gorged with blood, the surface of the brain red and injected, an infinite number of red spots in the medullary substance, serum in the ventricles and at the basis of the brain, &c.; for which, affusions of cold water on the head and trunk, sinapisms to the feet, the free use of leeches to the neck, and indeed all the therapeutic measures that seemed to be indicated, were employed without benefit; Dr. BLAUD was induced to see what would result from compression of the carotid arteries. (115) The results were very remarkable: the success in two cases, the only instances in which he had hitherto employed it, was complete. The compression, in one case, was continued during thirteen, seventeen, and twenty-five, seconds, after intervals of a period of several minutes. Each time, the patient recovered more or less from his state of stupor, and made some violent movements to disembarrass himself from the sensation which he seemed to experience from the compression of the neck; but, when this was interrupted, the stupor returned: after the third attempt, it did not recur. The compression was repeated every half hour, for about three hours, but only for the space of a few seconds each time, with a view to prevent a return of the disorder, by completely dissipating the little inordinate excitation that might remain. M. Blaud relates a similar case, which was communicated to him by one of his colleagues: the result was the same. The compression was here performed four different times; and was employed to remove a comatous affection consequent on an exposure of the bare head to the rays of an ardent sun. After the fourth compression, only a slight pain in the forehead remained, which was relieved before the following day by the application of some leeches to the temples.

Dr. Blaud's mode of executing the compression is thus described:

"We thrust the thumb and middle-finger of the right-hand between the carotid arteries and the sterno-mastoidean muscles, in the two correspondent regions at the lateral parts of the larynx. When the vessels were very evident by their pulsations, we approached them towards each other by contracting the fingers, and thus maintained them; and, in order that they might be more effectually compressed, we made the sides of the thyroid cartilage serve as points of resistance."

This practice is not, as Dr. Blaud supposes, novel; it was employed several years since by Dr. PARRY, of Bath, who published some observations on its efficacy in a Paper read to the Royal Society. *(See Philosophical Transactions,* 1811.*)*

The use of prussic acid (first introduced into practice by the

---

(115) *Observations sur l'Efficacité de la Compression des Artères Carotides, dans le Cas d'Engorgement sanguin du Cerveau, &c.* Par M. BLAUD, Médecin des Hopitaux de la Ville de Beaucaire. *Bibliothéque Médicale,* No. 284.

Italians, under the form of distilled laurel-water,) is now becoming somewhat general throughout the greater part of Europe, in various affections formerly termed nervous, but now known to depend on inflammatory excitement of some organ; as well as in several species of more evident chronic inflammation. As a general account of the mode of employing this remedy has already been inserted in this Journal, we shall now only give references to the observations which have been recently published, for the use of those who may desire to study them in a more particular manner. (116)

The use of *acupuncture*, a remedy derived from the Chinese physicians, appears to be getting introduced into practice in France. Dr. Haine, of Tours, (117) has published some observations demonstrative of its considerable efficacy in hysterical convulsions, chronic and vague rheumatic pains, &c.; and, when it is also considered that it has been thought of very favourably by Vicq-d'Azyr and Prof. Dupuytren, and has been much employed by Dr. Demours in various diseases of the eyes, we are led to notice it on the present occasion. (The method of performing it, used by M. Demours, will be described in the section on chirurgery.) In its mode of action, it appears to combine the efficacy of local blood-letting with very powerful counter-irritation; and may, therefore, probably be employed with benefit in many of the cases in which cupping, setons, and repeated vesicatories, have hitherto been resorted to.

Just as this sketch was completed, we received a work which will raise in a considerable degree the character of the United States as an academy for medical science: this is the *discourses on therapeutics and the materia medica*, of Dr. Chapman. (118) This work is strictly methodic, and constructed on principles worthy of an age in which medicine is professed to be cultivated with philosophic views. A few extracts, which we shall now transcribe, will furnish some ideas of the nature and character of the system of Dr. Chapman: the whole will engage our particular attention at a future period. Though it should here

---

(116) *De Præcipuis Acidi Prussici et Aquæ cohobatæ Laurocerasi Medicis Facultatibus, clinicis Observationibus comprobatis, specimen*, J. A. Manzoni, M.D. 4to. Patavii, 1818.

*Further Observations on the internal Use of the Hydro-cyanic (Prussic) Acid in Pulmonary Complaints, Chronic Catarrhs, Spasmodic Coughs, Asthma, Hooping-Cough, and some other Diseases; with full Directions for the preparation and administration of that Medicine.* By A. B. Granville, M.D. F.R.S. &c. 8vo. 1819.

*Einige Beobachtungen über die Anwendung der Blausaure in Nervenkrankheiten.* Von Dr. Ludike, arzt zu Breslau. In the Berlin *Journal der Practischen Heilkunde*, Jan. 1819.

(117) *Journal Universel des Sciences Médicales*, Jan. 1819.

(118) *Discourses on the Elements of Therapeutics and Materia Medica.* By N. Chapman, M.D. Professor of the Institutes and Practice of Physic and Clinical Practice in the University of Pennsylvania, &c. &c. 2 vols. 8vo. of 377 and 494 pages. 1817 and 1819. Webster, Philadelphia; and Souter, London.

be observed, that the doctrines of the author are not adduced as unobjectionable; or, individually considered, as totally novel. Some of them are consonant with those given in the commencement of this section, and there stated to have been the opinions of BICHAT; others, are somewhat analogous to those of DARWIN. We do not mention this with an intention to derogate from the merit of Dr. Chapman, but rather with the opposite intention; since the whole tenor of his work decisively evinces that they are really original with respect to himself, and he does not seem to be advised of the notions which Bichât had previously advanced. Dr. Chapman says,

"My theory of the operation of medicines is of modern date, and alleges, that they all act by exciting a local impression, which is extended through the medium of sympathy; that all changes in the condition of the fluids are wrought by impressions made through the intervention of the solids. To reach the circulation, medicines must pass either by the lacteals or lymphatics. Now, it seems more than probable, in either case, their powers would be so neutralized by the preparatory processes of animalization, as to be deprived of all activity. Whenever a medicinal substance is applied to a susceptible portion of the body, externally or internally, an action is excited, which is extended more or less, according to the diffusibility of the properties of the substance, or the degree of sympathetic connexion which the part may maintain with the body generally. Thus a set of actions is raised, every one of which is precisely similar, provided they are confined to the same system; by which is to be understood, parts of an identity of structure. If, however, the chain runs into other systems, it loses its homogeneous character; the actions being modified by the peculiar organizations of the parts in which they may take place."

Dr. Chapman, therefore, arranges all the materia medica under two classes,—local, and general or diffusible, stimulants. But, he remarks, "While I maintain so far the uniformity of the operation of medicines, I wish it to be understood, that I am not among those who, in the eagerness of generalization, have insisted that they are all endowed with the same properties, differing in degree of force, permanency, and diffusibility. I entertain, indeed, an entirely opposite view of the subject. My impression is, that scarcely any two agents produce entirely the same effects; and hence the infinitely diversified shades of disease, and the necessity for a variety of remedies in the management of them."

This classification of the materia medica was made some years since, when the author took the professor's chair in the university of Pennsylvania; but, he says,

"I am now persuaded, that a much more natural, as well as useful, arrangement of medicines might be made, on the principles of their affinities to the several systems of the body; and, should an opportunity be ever afforded me, it is the one which I shall attempt to establish."

Each of those propositions is a theorem, meriting long and deep reflection. Perhaps, the author has attributed more to sympathy than will be granted to it by physicians of the present age; though much of this reserve may arise from the consequences of early impressions on the mind. Dr. Chapman has probably written much solely for futurity.

For some very useful practical observations on the treatment of stricture of the anus, we refer to the memoir on this subject, inserted in the last volume of this Journal, by Mr. WHITE, of Bath.

§ VIII. From the mode in which we have treated of the different subjects relative to medicine, both as a science and as an art, it must be evident that but little can be adduced respecting *Chirurgery;* since no great improvement can now be expected to take place in it, as regards what is really designated by that term. The account given in the preceding sections of the subjects of physiology and pathology, must render it obvious that we shall here confine our observations to what appertains solely to *manual operations.*

Although the appropriating with accuracy the measures already in use, not the invention of new ones, is the chief object of the surgeon in this part of his medical agency, still there are some points yet undetermined relative to the method of executing those operations. This is especially the case in those applicable to aneurism, and other diseases of the vascular system. Some facts that have recently occurred to M. le Baron LARREY, (119) seem to show that the practice for the treatment of aneurisms in general, is by no means precisely determined. A brief account of those facts will render this sufficiently evident.

A man, about 30 years of age, was brought to the hospital "*Gros Caillou*," to be treated for an aneurismal tumor of the popliteal artery of the right limb. This tumor had continued stationary for several months, until very recently, when in a few days it acquired the size of two fists. All the veins of the limb were in a varicose state, and sanguineous effusion into the cellular structure of the limb had taken place. After consultation, Hunter's mode of operating was determined on, and performed. Pulsation ceased in the tumor immediately after the operation. Pressure was applied, to prevent the influx of blood into its cavity. In consequence of head-ache and sense of oppression

(119) *Bulletin de la Faculté de Médecine de Paris*, Jan. 1819.

about the precordia, the patient was thrice bled; after which, he became calm. The temperature of the limb, which had been inordinate, became equal with that of the rest of the body. The veins, which disappeared during the operation, and had subsequently attained an enormous size, (the great vena saphena being equal, in its lateral dimensions, to the little finger,) had now again become hardly apparent. During the three first days, the ligatures (120) were repeatedly tightened, because of slight pulsations having at different times been manifested in the tumor. The aneurism gradually diminished in bulk, and by the ninth day was reduced to half its former size: after this, it remained stationary. Supposing the artery to be now obliterated, the rolls of cere-cloth, which had been placed under the knots of the ligatures, were now removed: the wound then rapidly cicatrized. There was, then, in the centre of the aneurismal tumor, a fluctuation, which increased from day to day; and one night, after some slight exertion of the patient, the fluctuating point of the tumor burst, and gave vent to a large quantity of fluid, of the colour of wine-leys. M. Larrey laid the part open from one extremity to the other, and extracted from the aneurismal cavity an enormous mass of black and fetid coagulated blood. The cavity was filled with pledgets, moistened with a warm camphorated digestive; a retentive bandage was applied; and an analeptic regimen prescribed. Some days passed on without any remarkable occurrence. On the night of the 19-20th day, after the patient had taken wine and brandy to excess, violent hæmorrhage took place from the aneurismal cavity; and, before the attendant assistant-surgeon had arrived, the patient had lost nearly all his blood: he died in the ensuing night. This hæmorrhage M. Larrey supposes to have proceeded from one of the new arteries developed in the cyst. On dissection of the limb, the crural artery having been injected, this vessel was found converted into ligament, from the situation of the ligature to within a little distance of the aneurism. The state of putrefaction of the limb prevented any further examination.

This is one of the cases, M. Larrey thinks, in which the old operation should be preferred: that is, after having isolated and tied the popliteal artery immediately above the tumor, to open the sac and remove the coagulated blood, in order to prevent its decomposition, which took place in the above instance, and the hæmorrhage which thence resulted.

The subject of the preceding case was in a very weak and exhausted state, on his entry into the hospital; or, M. Larrey remarks, amputation of the limb would have been preferred to

(120) Two ligatures were applied to the artery, at a little distance from each other, in consequence of a collateral vessel having been wounded during the operation.

the operation that was performed. The opinions of SCARPA on this point, contributed to deter him from having recourse to that measure.

We shall here briefly notice an instance of the application of a ligature to the arteria innominata, for a supposed aneurismal tumor situated beneath the clavicles, which has been effected by Dr. VALENTINE MOTT, (121) of New-York: a detail of this case will be given in the ensuing number of this Journal. No remarkable accidents ensued from the operation, until the twenty-third day. On the twentieth, the patient was so well as to be able to walk down stairs; but, on the twenty-third, on turning in bed, hæmorrhage, to about twenty-four ounces, took place from the situation of the ligature. Repeated hæmorrhages ensued from this time; and, on the twenty-fifth day from the operation, the patient died. Dissection showed that the bleeding had depended on ulceration of the artery in the situation of the ligature: a probe would pass into this from the trunk of the innominata.

Another favourable instance of the application of a ligature to the external iliac artery, performed by Mr. BOND, of Norwich, was related in the last volume of this Journal. Mr. KIRBY, of Dublin, has also recently executed it, with complete success. (122)

Prof. DUPUYTREN presented a patient before a sitting of the Faculty of Medicine, in November last, who had been cured of popliteal aneurism in fifteen days, by compression on the lower part of the femoral artery, made by means of a compressor formed of two-thirds of a circle; having at one extremity a fixed, and at the other a moveable, pad; a contrivance by which the force of the pressure could be varied and regulated without the risk of entirely removing it, which might be the means of interrupting the process necessary to effect the cure. This is the second that M. Dupuytren has cured by the same means.

The mode of performing *acupuncture*, used by Dr. DEMOURS, is now to engage our attention. When employing it for some of the varieties of ophthalmia, Dr. Demours has effected it in the infra-spinalis muscle, choosing the part where the muscle has the greatest thickness, which is just beneath the spine

(121) *The Medical and Surgical Register; consisting chiefly of Cases in the New-York Hospital.* By JOHN WATTS, jun. M.D. VALENTINE MOTT, M.D. and ALEXANDER H. STEVENS, M.D. 8vo. pp. 163. Collins and Co. New-York; and Souter, London. 1818.

(122) *Cases, with Observations on Wry-Neck; on the Reduction of Luxation of the Shoulder-Joint; on the Operation for Hare-Lip; on Cartilaginous Substances in the Knee-Joint; on Aneurism, &c. &c.* By JOHN KIRBY, A.B. Member, and one of the Censors, of the Royal College of Surgeons, in Ireland; Surgeon to St. Peter's and St. Bridget's Hospital; and Lecturer on Anatomy and Surgery. 8vo. pp. 166. Burgess and Hill, London. 1819. The other subjects of this work will be considered at a future time.

of the scapula. A cupping-glass is applied, in the ordinary way; and, as soon as this is removed, several needles (for example, five or six,) are to be plunged perpendicularly, in succession, towards the centre of the part which had been covered by the glass: the needles should be inserted by rolling them, in a manner, between the thumb and fore-finger. Common sewing needles, of from two to two inches and a half in length, are proper for the purpose; their points should be very sharp, and they should have sufficient strength to avoid the danger of their being broken: they may penetrate to the depth of about nine lines in a person whose shoulder is tolerably fleshy, and it may be prudent to cover them with wax beyond this extent. If they are let remain in for three or four minutes, as is usual, the patient should be cautioned not to move either the arm or shoulder.

Several tracts have been recently published on the mode of forming an *artificial pupil;* but an history of the methods proposed cannot here be given: and, each operator considering his own the best, it is hardly possible to form any precise conclusion from a consideration of their various works on the subject: we shall, therefore, merely give references to them. (123)

§ IX. The act of parturition being in itself the result of so peculiar a series of functions; and forming also, in a great measure, so distinct a subject for the application of the medical art; we have chosen to consider what immediately relates to those objects in a particular section: it has also appeared to us to be advisable, that such diseases as seem to be expressly consequent on the puerperal state, should be comprised in the same view. These are all equally the subjects of the care of the practitioner in obstetricism.

Several of the most important treatises and observations on these subjects, that have been produced within the last semi-annual period, have already been either extensively considered or wholly inserted in the last volume of this Journal; as, those of Prof. FODERÉ, (124) Mr. KING, (125) our correspondents, Mr. HAMILTON (126) and Dr. H., and that of Mr. POWER. (127)

---

(123) *Further Account of the Result of an Operation for performing artificial Pupil.* By J. P. MAUNOIR. *Medico-Chirurgical Transactions,* Vol. IX. Part II. Longman and Co. London.

*Observations on the Operation for Artificial Pupil.* By E. RYAN, M.D. *Dublin Hospital Reports,* Vol. II.

*Commentatio de Coresmorphosi, &c.* Auctore GUILIELMO WAGNER, M.D. Gottingen. 8vo. pp. 80.

*De Raphiankistro, &c. &c.* Von J. EMDEN, M.D. &c. &c. 8vo. pp. 38. Gottingen.

*Ueber den Gegenwärtigen Zustand der Künstlichen Pupillenbildung in Deutschland.* Von W. A. J. SCHLAGINTWEIT, Dr. der Med. und Chir. &c. 8vo. VIII. und 117 seit. Munchen.

(124) *Medical and Physical Journal,* vol. xli. pp. 350, 457, 526.

(125) *Ibid.* p. 512. (126) *Ibid.* p. 121. (127) *Ibid.* p. 332, 424.

The only treatises which come within the view taken for the arrangement of this essay, are those of Dr. Dewees, (128) of Philadelphia, and Dr. Douglass, (129) of Dublin.

The closing limits of this sketch oblige us to defer entering into the consideration of the work of Dr. Dewees until a subsequent period. This physician, it may however be at present remarked, considers that parturition is not a painful function in the human female in the uncivilized state; and in the present essay he endeavours to ascertain the cause of the change we now witness. Mr. Power has, we consider, commenced the investigation of this interesting question in a very judicious manner: he has traced, with much accuracy and precision, the nature of the results; and it is by proceeding upwards from these phenomena, that we can alone expect to arrive at the knowledge of their causes. The work of Mr. Power must be considered as one of the particularly remarkable literary productions of the existing period.

Considering the "Explanation" of Dr. Douglass in a theoretical manner, it appears calculated to produce important benefit to the practice of obstetricism. The description which he has given of the mode in which the evolution of the fœtus takes place in original presentations of a shoulder, seems to render it evident that such an occurrence may occur, when left to nature, much oftener than has been generally supposed. Dr. Douglass observes, that, in all the recorded cases, it is stated that, shortly before this evolution took place, the shoulder of the child has been forced very low into the pelvis; and that the thorax had occupied so much of its cavity as to preclude the practicability of the hand being passed up into the uterus for the purpose of turning, as is usually done in such presentations. The explanation of the evolution given by Dr. Denman, was, that, by the pressure of the fundus of the uterus on the breech of the child, its body was made to turn on its own axis; the thorax rising up into the uterus as the breech was expelled from it, until at length the latter actually became the presenting part. The possibility of such an occurrence is doubted by Dr. Douglass: his own observations have led him to determine, that the shoulder and thorax are, at every successive pain, forced still lower in the pelvis, until the ribs of the side, corresponding with the presenting arm, press on the perineum. At this pe-

---

(128) *An Essay on the Means of lessening Pain, and facilitating certain Cases of difficult Parturition.* By W. P. Dewees, M.D. Lecturer on Midwifery in Philadelphia, &c. 8vo. pp. 156. Dobson and Son, Philadelphia; and Souter, London. 1819.

(129) *An Explanation of the real Process of the "Spontaneous Evolution of the Fœtus,"* &c. By John C. Douglass, M.D. &c. 8vo. pp. 45. Hodges and M'Arthur, Dublin. 1819.

riod, not only the entire arm, but the shoulder also, can be perceived externally, with the clavicle lying under the arch of the pubis. By further uterine contractions, the ribs are forced more forward, appearing at the os externum as the vertex would in a natural labour; the clavicle having been, by degrees, forced round on the anterior part of the pubis, with the acromion facing the mons veneris. The fœtus now forms the larger segment of a circle, with the head resting on the pubis internally, and the breech in the hollow of the sacrum, or at the brim of the pelvis ready to descend into it; and, by a few more contractions of the uterus, this, and the remainder of the trunk and the lower extremities, are expelled. Thus, the breech is not the first part of the body which appears at the os externum: it is the side of the thorax, corresponding with the presenting arm, which thus appears; but the breech is momentarily expelled before the opposite side of the thorax. The head and one arm still remain. The presenting arm now gets forward on the symphisis pubis *externally*, and the head and other arm are distending the perinæum and the os externum.

The above description is derived from observations made in seven cases.

From the above rationale of the process of the evolution, it is evident that it is an occurrence which may rationally be expected, and which may also be calculated on, under certain circumstances; whilst, according to the explanations previously given of it, the occurrence of it must appear a matter of such uncertainty, as to lead the practitioner to the use of almost any other measures, on which more confidence of a favourable issue could be placed. The application of this consideration to practice, is sufficiently obvious. It is not meant that it should exclude the necessity of turning in those cases in which that operation has generally been considered to be indicated; but only under the following circumstances: "If the arm of the fœtus should be almost entirely protruded, with the shoulder pressing on the perinæum; if a considerable portion of the thorax be in the hollow of the sacrum, with the axilla low in the pelvis; if, with this disposition, the uterine efforts be still powerful, and if the thorax be forced sensibly lower during the presence of each successive pain:" for then, the evolution may with great confidence be expected; and an operation, always terrifying, and commonly dangerous, to the suffering female, may be avoided.

On concluding an essay, intended to give such a view of the recent events in the universal republic of medicine as may disclose to the reflective mind some knowledge of those occurrences of the most importance, both with respect to their immediate influence, and the future revolutions they are qualified to produce; we are led to offer some remarks, dictated by a sense of duty to our own exertions, and by a proper regard for the interests of the science to which they have been devoted.

When the great extent of the subjects to be comprised in the series of dissertations commencing with the present, is duly reflected on; and when it is also considered, that, according to the method traced in the preceding essay, they must be the results of individual researches; it will be evident that repeated efforts will be requisite to give them the degree of perfection to which they may be raised by the same talents and similar exertions, with those which were employed in the construction of that here produced. Progressive experience will probably suggest means by which the arrangement of the various subjects may be rendered more lucid, and by which a more numerous assemblage of facts may be embraced in an equal extent of literary narration. Several retrospective observations and adventitious remarks, which have here been adduced in order to show precisely the course we intend to pursue, and to obviate the obscurities which might attend a novel arrangement of the subjects of medical history, will be avoided as unnecessary on future occasions. Experience will also be requisite to enable us to seize, in the course of our literary researches, all the objects dispersed in divers tracts, often concealed under fictitious appearances, and to display them in their proper characters, and arrange them in their appropriate order. Time will also be necessary to render fully effective the measures which have been taken to acquire information of all that may transpire in the more remote parts of the globe.

As an apology for any rudeness which may appear in these essays, relating especially to their literary character, we observe, that the rapidity with which they must be written, necessarily prevents the application of what the finest of critics stated to be requisite, in order to give to a literary production the faintest reflection of those traits, which he has wholly appropriated to his own works: this, however, is not worthy of consideration in the estimation of their real merits; for medicine is in itself too interesting to every reflective mind, to render the borrowed ornaments of rhetoric, or refined elegance of diction, at all desirable in the language in which its dictates are conveyed. Leaving to the votaries of the gay train of the Muses, their Lydian sportiveness, their flowery garlands, and their crowns of bays; the followers of HIPPOCRATES will endeavour to assume the simple gracefulness of manner which he inculcated by precepts, and exemplified in himself.

*London; June the 30th,* 1819. W. HUTCHINSON.

